# *Entamoeba* and *Giardia* parasites implicated as hosts of CRESS viruses

**DOI:** 10.1038/s41467-020-18474-w

**Published:** 2020-09-15

**Authors:** Cormac M. Kinsella, Aldert Bart, Martin Deijs, Patricia Broekhuizen, Joanna Kaczorowska, Maarten F. Jebbink, Tom van Gool, Matthew Cotten, Lia van der Hoek

**Affiliations:** 1 Laboratory of Experimental Virology, Department of Medical Microbiology and Infection Prevention, Amsterdam UMC, University of Amsterdam, Meibergdreef 9, 1105 AZ Amsterdam, The Netherlands; 2 Laboratory of Clinical Parasitology, Department of Medical Microbiology and Infection Prevention, Amsterdam UMC, University of Amsterdam, Meibergdreef 9, 1105 AZ Amsterdam, The Netherlands; 3 MRC/UVRI & LSHTM Uganda Research Unit, 3FC6+Q3 Entebbe, Uganda; 4 MRC-University of Glasgow Centre for Virus Research, G61 1QH Glasgow, UK

**Keywords:** Phylogenetics, Parasite genomics, Viral evolution, Viral host response

## Abstract

Metagenomic techniques have enabled genome sequencing of unknown viruses without isolation in cell culture, but information on the virus host is often lacking, preventing viral characterisation. High-throughput methods capable of identifying virus hosts based on genomic data alone would aid evaluation of their medical or biological relevance. Here, we address this by linking metagenomic discovery of three virus families in human stool samples with determination of probable hosts. Recombination between viruses provides evidence of a shared host, in which genetic exchange occurs. We utilise networks of viral recombination to delimit virus-host clusters, which are then anchored to specific hosts using (1) statistical association to a host organism in clinical samples, (2) endogenous viral elements in host genomes, and (3) evidence of host small RNA responses to these elements. This analysis suggests two CRESS virus families (*Naryaviridae* and *Nenyaviridae*) infect *Entamoeba* parasites, while a third (*Vilyaviridae*) infects *Giardia duodenalis*. The trio supplements five CRESS virus families already known to infect eukaryotes, extending the CRESS virus host range to protozoa. Phylogenetic analysis implies CRESS viruses infecting multicellular life have evolved independently on at least three occasions.

## Abstract (web summary)

Metagenomics allows virus genome discovery, but the viral hosts are often not identified. Here, Kinsella et al. use recombination events between virus genomes, statistical association of viruses to hosts in clinical samples, and analysis of endogenous viral elements in host genomes to identify probable hosts of three CRESS virus families.

## Introduction

Determining hosts of viruses is integral to understanding their medical or ecological impact. This is particularly challenging for virus species discovered using metagenomic sequencing, since samples such as stool or environmental matrices contain diverse potential hosts^1,2^. A decade of metagenomic studies have shown that viruses with circular Rep-encoding single-stranded DNA genomes (CRESS viruses) are highly diverse and pervasively distributed^3,4^, yet currently, the majority of known CRESS virus genetic diversity falls outside established families with characterised hosts^5^. Five CRESS virus families have experimentally confirmed eukaryotic hosts: *Bacilladnaviridae*, *Circoviridae*, *Geminiviridae*, *Genomoviridae*, and *Nanoviridae*^6^, respectively infecting diatoms^7^, vertebrates^8,9^, plants^10^, fungi^11^ and plants^12^. Unclassified lineages of metagenomically identified CRESS diversity exist in at least six further clusters labelled CRESSV1 through CRESSV6, and a multitude of chimeric species difficult to place phylogenetically^13^.

Unclassified CRESS viruses are frequently found in human and non-human primate stool samples, generating interest into their host specificity and potential impact on health^14–17^. Classically, virus–host relationships are determined via recognition of host disease, followed by virus isolation in cell culture. Since this is impractical for metagenomically identified viruses, case-control studies are used to reveal associations between viruses and disease. Importantly though, this does not confirm the host; for example, the CRESS virus family *Redondoviridae* is associated with human periodontal disease and critical illness^18^, but it remains unknown whether the viruses infect humans or a separate host, itself associated with or causing the observed clinical outcomes.

Genomic evidence of virus–host interactions can directly establish links between species. For instance, the *Smacoviridae*, a CRESS virus family previously assumed to infect eukaryotes, were recently suggested to infect archaea^19^ on the basis of CRISPR spacer sequences matching a smacovirus inside the genome of an archaeon. Similarly, virus genomes can integrate into host genomes, leaving endogenous viral elements, identification of which reveals historical infections^20,21^. Searches for endogenous viral elements related to CRESS viruses have revealed integrations into the genomes of eukaryotes, for instance, sequences related to the replication-associated protein (Rep) of *Geminiviridae*, major global crop pathogens, are integrated in the tobacco genome^22^.

Rep-like sequences are found in the genomes of the protozoan gut parasites *Entamoeba histolytica* and *Giardia duodenalis*^23^, important human pathogens belonging to distantly related genera^24^. The Rep-like elements could imply that the parasites host CRESS viruses, however, the sequences do not belong to a known family^3^. One proposed alternative hypothesis is that that they were gained from bacterial plasmids directly^23^, which are thought to be the ancestors of CRESS virus *Rep* genes^25^. Compatible with this, no sequence related to a capsid protein (Cap) has been found integrated in *Entamoeba* or *Giardia* genomes. While several studies have discussed or attempted to identify an association between CRESS viruses and gut parasites^3,26–28^—none has been found to date—and indeed no CRESS virus is known to infect any protozoan. Here we provide evidence that the parasite genera *Entamoeba* and *Giardia* are hosts of CRESS viruses, introducing a framework for host determination of metagenomically sequenced viruses that can be widely applied.

## Results

### Unclassified CRESS viruses are associated to parasites in human stool

Stool samples from 374 individuals (belonging to two independent cohorts, see "Methods") were enriched for viruses using the VIDISCA method, metagenomically sequenced, and bioinformatically analysed to identify unknown CRESS viruses. We used sequence assembly of short reads in combination with inverse PCR and Sanger sequencing to determine 20 full-length CRESS virus coding sequences (accessions MT293410.1–MT293429.1). The 20 sequences included 18 complete genomes covering all untranslated regions, and these had a genome organisation akin to known CRESS viruses, with a conserved nonanucleotide motif at an apparent replication origin, and open reading frames that aligned to viral *Rep* and *Cap* genes (Supplementary Table 1). Using PCR or mapping of sequencing reads to the assembled genomes, we determined that 21 of 374 samples were positive for the viruses.

All 374 samples were also analysed for the presence of *Entamoeba* and *Giardia* parasites using either microscopy, sequencing-based approaches, PCR targeting the 18S ribosomal RNA, or a combination thereof (see “Methods”). We observed that all 21 of the samples containing one of the CRESS viruses were also positive for either *Entamoeba* or *Giardia* (Table 1 and Supplementary Table 2). Across the 374 samples, presence of any of the 20 viruses was significantly associated with *Entamoeba* or *Giardia* infection using Pearson’s chi-squared test (*χ*^2^ = 36.77, *p* < 0.001), therefore we hypothesised that the viruses infected one or both of the parasites. To test the possible host role of other gut protozoa (including *Blastocystis*, *Dientamoeba*, *Cryptosporidium* and *Endolimax* among others), we carried out further parasitological typing on the 21 virus-positive samples (see “Methods”). We found these taxa were absent from all, or a majority of the 21 samples—implying they are not hosts of the viruses (Supplementary Table 2).

**Table 1:** *Entamoeba* and *Giardia* status of human samples positive for any of the CRESS viruses identified in this study.

Parasite status	Number of samples (*n* = 374)	Positive for CRESS viruses identified in this study
*Entamoeba* positive only	130	18
*Giardia* positive only	3	0
*Entamoeba* and *Giardia* positive	8	3
*Entamoeba* and *Giardia* negative	233	0

### Whole CRESS virus genomes are integrated into parasite genomes

**Fig. 1:**
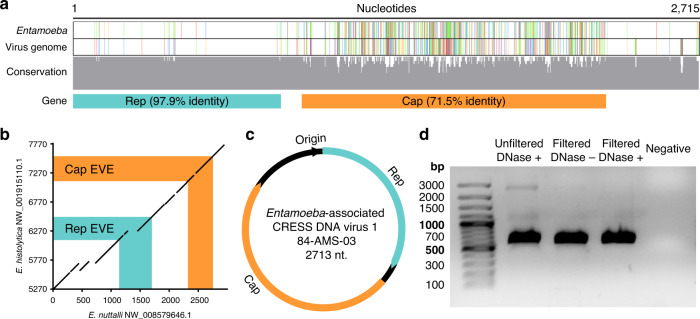
Whole CRESS virus genomes are integrated in Entamoeba genomes. **a** Cropped nucleotide alignment between *Entamoeba dispar* contig (AANV02000527.1) containing a complete virus integration and the genome of *Entamoeba*-associated CRESS DNA virus 1, isolate 84-AMS-03 (accession MT293413.1); also see Supplementary Fig. 2. Coloured vertical bars denote single nucleotide variations between the sequences (adenine = green, guanine = red, thymine = blue, cytosine = orange), with conservation across the alignment displayed below. **b** Dotplot of BLAT generated nucleotide alignment between endogenous viral elements and flanking sequence from two closely related *Entamoeba* species (*x*-axis sequence reverse complemented). **c** Example of the circular genome organisation of identified CRESS viruses. **d** Exogenous virus DNA is protected by a viral capsid, as it can be PCR-amplified after filtration and treatment with DNase (one independent experiment).

In order to identify endogenous viral elements related to the identified CRESS viruses, we aligned all 20 coding sequences to GenBank databases, namely the non-redundant nucleotide (BLASTn, Supplementary Table 3), protein (BLASTx, Supplementary Table 4), and whole-genome shotgun contigs of *Entamoeba* and *Giardia* (BLASTn, Supplementary Table 5). Viral queries aligned with high identity and coverage to nucleotides and predicted proteins from parasite genomes, suggesting the presence of CRESS virus-derived endogenous viral elements. The 20 viruses were not uniform in their database hits, showing genetic variation among them; each virus strongly aligned to sequences from either *Entamoeba* or *Giardia*, but not both, suggesting the presence of distinct viral lineages with independent virus–host relationships. Among viruses aligning to sequences from the *Entamoeba* genus, variability was also observed in the parasite species—queries either hit *E. histolytica*, *E. dispar*, *E. nuttalli*, or *E. invadens*. Among viruses aligning to sequences from *Giardia duodenalis*, alignments were found against major genotypes infecting humans, specifically A2 and B. Importantly, alignment to parasite genomes revealed evidence of whole virus genome integrations. For example, one virus genome (accession MT293413.1) aligned inside an 11.6 kilobase (kb) contig from *E. dispar* (AANV02000527.1) with 100% query coverage and 84% nucleotide identity (Fig. 1a), while another (accession MT293421.1) aligned inside a 15.2 kb contig from *G. duodenalis* (AHGT01000120.1) with 99% query coverage and 73% nucleotide identity. As the only known examples of parasite endogenous viral elements containing both the *Rep* and *Cap* viral genes, they cast doubt on the hypothesis that Rep-like elements in protozoal genomes were derived from bacteria^23^. Since CRESS virus integration is likely mediated by the Rep protein during viral genome replication in the host nucleus^29^, the elements directly implicate *Entamoeba* and *Giardia* as hosts.

We next considered and eliminated potential sources of error, firstly, that parasite genomes did not truly contain CRESS endogenous viral elements, but rather that the assemblies were contaminated with virus genome sequences found in the original sample or reagents. To eliminate this possibility, we compared independently generated genome assemblies of *E. histolytica* and *G. duodenalis*, which were derived from parasite stocks in different laboratories or biobanks, and included strains isolated from patients across multiple countries and years. We could identify the same endogenous viral elements in several of the assemblies, for example an element (EMD43492.1) from *E. histolytica* strain KU27, isolated in Japan in 2001, was also found in strain HM-3:IMSS, isolated in Mexico in 1972 (100% coverage, 100% sequence identity), and three independent assemblies of strain HM-1:IMSS, isolated in Mexico in 1967 (100% coverage, 99.9% sequence identity, Supplementary Tables 6 and 7). Furthermore, in one case an element and its flanking sequence could be aligned between the closely related species *E. histolytica* and *E. nuttalli* (Fig. 1b). This provides evidence of a shared viral integration that must have originated prior to host speciation, although the date of this divergence is currently unknown. Interestingly, *G. duodenalis* elements displayed a lineage-specific distribution, found universally in assemblies of lineages A2 and B, but absent from lineage A1 assemblies and the lone assembly of lineage E (Supplementary Table 7). The results suggest population-level fixation of elements in specific parasite lineages, rather than contamination leading to a misassembly. To rule this out however, for *E. histolytica* HM-1:IMSS we closely examined raw sequencing coverage across a selected endogenous viral element and its flanking sequence, showing that Sanger sequence reads span the element with no coverage aberrations (Supplementary Fig. 1A). We secondarily confirmed this by analysing the raw reads of strain KU27, isolated over thirty years later, with consistent results (Supplementary Fig. 1B). For *G. duodenalis* we examined the elements present in a recent reference quality assembly (GCA_011634595.1, isolate GS, lineage B), since this was generated using a combination of conventional short-reads and nanopore long-reads^30^. The latter technology vastly improves the scaffolding and repeat-resolution of assemblies, and confirmed the presence of endogenous viral elements within host sequence, even resolving a 10 kb-long tandemly repeated element not previously detectable in assemblies relying on short-read technology alone (Supplementary Fig. 1C). For further evidence that the endogenous viral elements were a true genomic feature, we looked for a small RNA response against them in *E. histolytica*, since the parasite silences its own genes post-transcriptionally via the RNA interference pathway^31^. We utilised public data comprising small RNAs immunoprecipitated in association with AGO2-2^32^, which is the component of the RNA interference pathway responsible for binding RNA guide strands and target mRNA cleavage, mapping the small RNAs to *E. histolytica* contigs containing endogenous viral elements (Supplementary Fig. 2). We found small RNA coverage peaks coinciding with several endogenous viral elements, including one known to be transcriptionally active^33^, suggesting host silencing of the elements. A notable but untested implication is that mRNAs from exogenous CRESS viruses infecting *E. histolytica* may also be silenced by such a response, which may therefore function in antiviral defence, since some small RNA sequences also had exact matches to the CRESS virus sequences of our study (Supplementary Fig. 2).

We secondly confirmed that viral genomes identified in human clinical samples were derived from exogenous viruses, since an alternative possibility is that they represented endogenous viral elements sequenced from parasite chromosomal DNA. The likelihood of this occurrence was minimised by the VIDISCA sequencing library preparation, which included removal of cell debris and degradation of residual chromosomal DNA via DNase treatment, however, for confirmation, we visually inspected viral reads to verify sequence overlap at the beginning and end of contigs. In this way, we could establish that the majority of viral coding sequences found in human samples were circular whole genomes (*n* = 18, Fig. 1c), and therefore were not from a larger sequence context such as a parasite chromosome. Finally, since exogenous viruses are small in comparison to eukaryotic cells, and their genomes are encapsidated in a protein shell, we experimentally confirmed these features. We filtered supernatant from virus-positive faecal suspension through 1200 and 200 nm pores, and treated the filtrate with DNase to remove unprotected DNA, finding that viral DNA could still be amplified by PCR (Fig. 1d). This shows that the genetic material was protected by a structure, most likely a capsid.

### Protozoa-infecting viruses are from previously unknown families

**Fig. 2:**
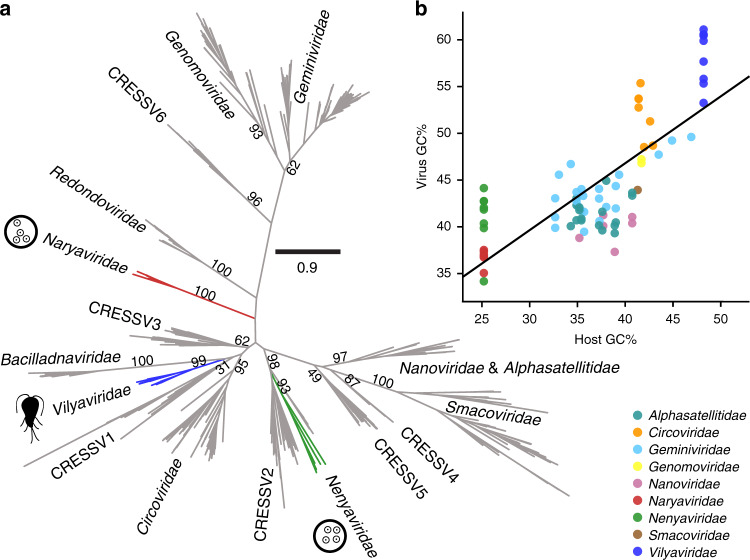
Parasite-infecting CRESS virus genomes are distinct from known CRESS diversity. **a** Phylogenetic maximum-likelihood tree of the Rep protein, scale bar refers to amino acid substitutions per site, numerical values represent bootstrap support of major nodes. The *Naryaviridae*, *Nenyaviridae*, and *Vilyaviridae* contain endogenous viral element sequences extracted from host genomes, respective pictograms of *Entamoeba* (tetranucleate cyst stage) and *Giardia* (flagellated trophozoite stage) are shown to indicate this. Five public viral genomes were also found to cluster within these families (MG571899.1, KU043415.1, MH617639.1, KY487991.1 and LC406405.1). **b** Virus GC-content positively correlates with host GC-content (linear regression, *n* = 79 biologically independent viral genome sequences, *r*^2^ = 0.58, *p* = 0.01).

Virus alignments to endogenous viral elements in parasite genomes already suggested that distinct viral lineages with independent virus–host relationships were present among the sequences. We, therefore, resolved the relationships of the exogenous viruses by building a maximum-likelihood phylogenetic tree of the Rep protein. Sequences extracted from Rep-like endogenous viral elements in *Entamoeba* spp. and *G. duodenalis* were included to identify their closest relatives and reveal which virus lineages were the original donors. Known CRESS virus diversity was incorporated by modifying a previously published chimaera-free Rep protein database of CRESS virus families and clusters^13^. We included the *Redondoviridae* in the dataset in addition to our own sequences and the closest viral relatives of our 20 sequences identified by BLAST searches. The viruses belonged to three strongly supported monophyletic Rep lineages, all phylogenetically positioned outside known families (Fig. 2a). Protein sequences from parasite endogenous viral elements clustered within each of the three lineages, and never outside, a firm indication that the exogenous virus lineages were the original donors of the endogenous viral elements. Notably, *Entamoeba* endogenous viral elements clustered exclusively within two of the three lineages, while *Giardia* endogenous viral elements only clustered with the third, indicating their different host specificity. Since the lineages do not belong to a known CRESS virus family, we propose the establishment of three virus families to house them. Following the practice of naming CRESS virus taxa with reference to their circular genomes, we suggest naming the families after three rings from Tolkien’s canon: *Naryaviridae* and *Nenyaviridae* for the two *Entamoeba*-infecting virus families and *Vilyaviridae* for the *Giardia* infecting family. The three families are phylogenetically distributed among known CRESS virus diversity, and imply that lineages infecting multicellular life evolved on at least three independent occasions, namely (1) the lineage including *Geminiviridae* and *Genomoviridae*, (2) the *Circoviridae*, and (3) the *Nanoviridae*. The *Nenyaviridae* are nested within the CRESSV2 cluster, suggesting these viruses may also infect protozoa.

We delimited CRESS virus genera using a cutoff of 50% Rep protein identity, following a recent literature example^18^. Genera infecting the same host genus were assigned a Greek number and named with reference to the host (ent for *Entamoeba* and gia for *Giardia*) (Supplementary Table 8). The *Naryaviridae* were thus divided into two genera (Protoentvirus and Deuteroentvirus), *Nenyaviridae* into two (Tritoentvirus and Tetartoentvirus), and *Vilyaviridae* into three (Protogiavirus, Deuterogiavirus, and Tritogiavirus). Although the viruses display large intra-family sequence diversity, the families do share distinctive features: *Naryaviridae* and *Nenyaviridae* genomes have sense open reading frames, while *Vilyaviridae* genomes have either ambisense or antisense open reading frames. Nucleotide usage measured by GC-content varies within each of the three families, but *Naryaviridae* and *Nenyaviridae* have on average 37% and 42% respectively, while the *Vilyaviridae* have a high 59%. The GC-contents of *Naryaviridae* and *Vilyaviridae* respectively represent low and high extremes among eukaryotic CRESS viruses. Since a positive association between host nucleotide usage and virus nucleotide usage has previously been observed among single-stranded DNA bacteriophages^34^, we hypothesised that this also underlay the observed distribution. To test this, we modelled the GC-content of CRESS virus lineages against those of known or proposed hosts using linear regression (Fig. 2b and Supplementary Table 9). For *Entamoeba* and *Giardia* we used the GC-content of *E. histolytica* (25.2%, assembly GCA_000365475.1) and *G. duodenalis* (48.2%, assembly GCA_000498735.1), respectively. A positive association was found between virus and host nucleotide usage (*r*^2^ = 0.58, *p* = 0.01), consistent with the proposed virus–host relationships. The association may be due to codon usage bias, wherein virus codon usage is constrained by host transfer RNA availability^35^. Despite the positive association, exogenous viruses from the three families did have a higher GC-content than their hosts by an average of 12.6%, suggesting the existence of additional selection pressure on GC-content counter to that of transfer RNA mediated protein translation efficiency. In contrast with exogenous viruses, endogenous representatives of each family had a reduced GC-content, in some cases closely resembling that of the host (Supplementary Fig. 3). We hypothesise that this is due to genetic drift resulting from relaxed selection on elements after integration, wherein the oldest elements may have the lowest GC-content.

### Viral recombination networks identify virus–host clusters

**Fig. 3:**
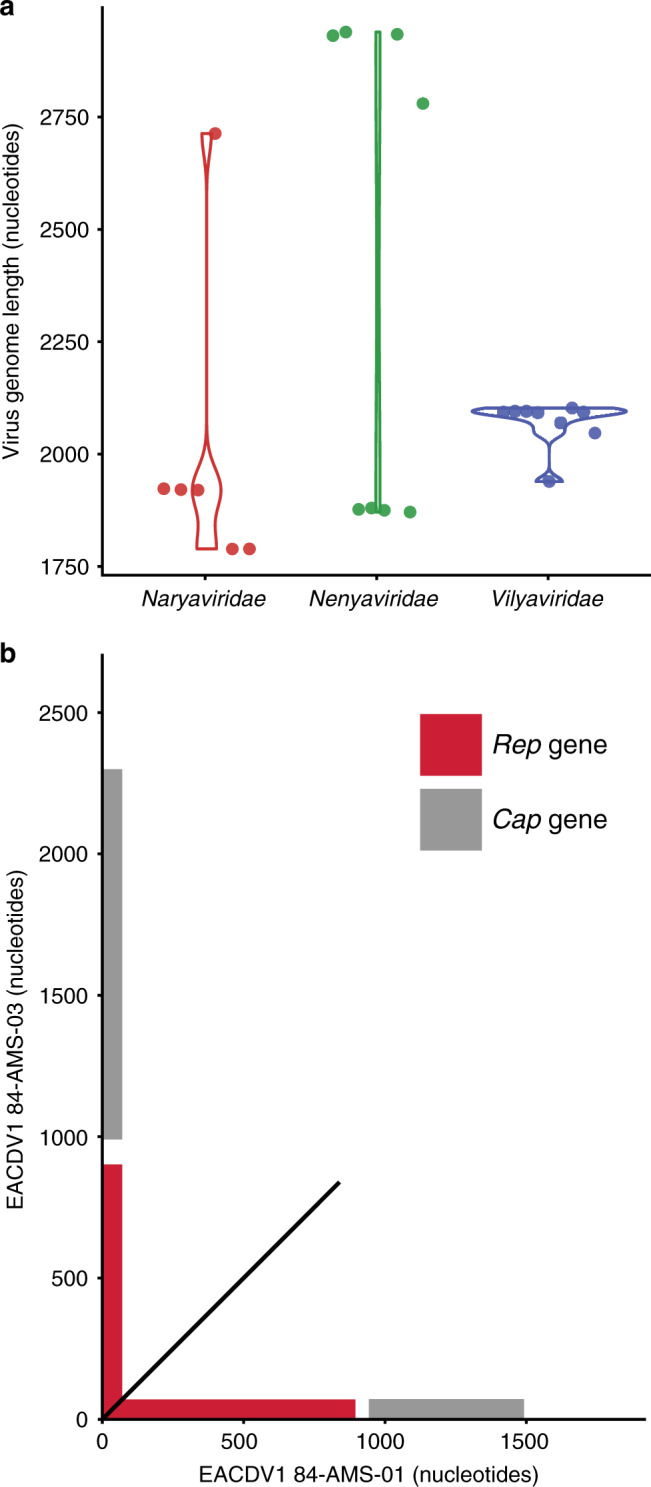
*Cap* genes of different ancestry in *Naryaviridae* and *Nenyaviridae*. **a** Genome length variation in *Naryaviridae*, *Nenyaviridae*, and *Vilyaviridae*. Eighteen complete CRESS virus genomes identified in this study were plotted alongside five complete publicly available genomes. **b** Dotplot of BLAT generated nucleotide alignment between a short and a long genome from the *Naryaviridae*, showing no detectable alignment between the *Cap* genes.

During genomic analysis of the CRESS viruses we observed a striking bimodal genome length distribution in both *Naryaviridae* and *Nenyaviridae*, but not in *Vilyaviridae* (Fig. 3a). BLAT alignment between two *Naryaviridae* genomes from the ends of the length distribution showed that the irregularity was caused by *Cap* genes of different lengths (averaging 179 and 439 amino acid residues respectively) with no detectable nucleotide sequence similarity, while the *Rep* genes were closely related (Fig. 3b). The two Cap proteins also had no detectable protein sequence identity upon pairwise BLASTp analysis, suggesting that the smaller of the two is not simply a partial protein, but a protein of different ancestry. To ensure that this was not a result of genome misassembly, we confirmed that Sanger sequencing reads overlapped both the *Rep* and *Cap* genes. Different ancestry of *Cap* genes found in combination with a *Rep* gene strongly suggested recombination of complete genetic modules (i.e. replicative and structural genes). Recombination between viruses occurs during genome replication within the host, and evidently the host range of a virus dictates its potential recombination partners^36^. Detection of recombination between viruses can therefore be used to group together viruses into virus–host clusters.

**Fig. 4:**
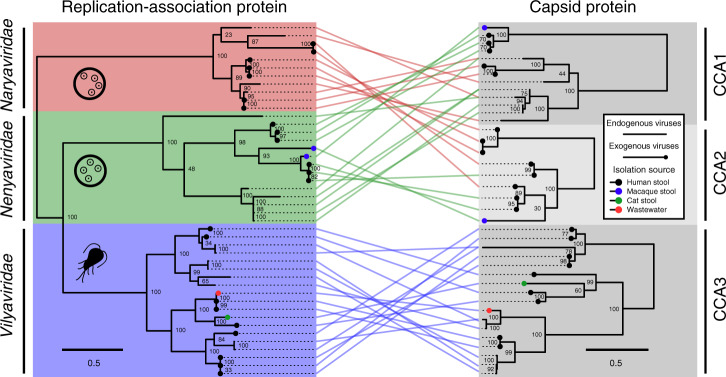
Recombination of genetic modules between virus families infecting the same host. Phylogenetic maximum-likelihood trees of viral Rep and Cap proteins, scale bars refer to amino acid substitutions per site, numerical values represent bootstrap support. Lines connect genes from the same virus or physically close endogenous viral genes. Pictograms of *Entamoeba* (tetranucleate cyst stage) and *Giardia* (flagellated trophozoite stage) are shown to indicate virus host. CCA = CRESS virus *Cap* assemblage.

To investigate recombination among the identified CRESS viruses, we constructed maximum-likelihood phylogenetic trees of Rep and Cap protein sequences from the three viral families, also including endogenous viral elements if *Rep* and *Cap* genes were found in close proximity in the protozoal genome (Fig. 4). Since *Cap* genes could not be globally aligned together, we first separated them into similar protein clusters which were then aligned and analysed individually. The Rep proteins were resolved into the three groups previously observed, corresponding with the three viral families. The Cap proteins were also divisible into three clusters, and we subsequently refer to these as CRESS virus *Cap* assemblages (CCAs). We visualised gene swapping between lineages by linking proteins extracted from the same genome across the two phylogenies, and this uncovered clear evidence of recombination of genetic modules between the *Naryaviridae* and *Nenyaviridae*. Members of these *Rep* families possessed either CCA1 (averaging 467 amino acid residues) or CCA2 (averaging 180 amino acid residues), with all four possible *Rep* and *Cap* gene combinations represented. Importantly, while evidence of recombination was also visible within the *Vilyaviridae*, they always possessed CCA3, therefore no evidence for recombination between *Vilyaviridae* and members of either the *Naryaviridae* or *Nenyaviridae* was found. The data strongly support the proposed host-range of the viruses, specifically *Naryaviridae* and *Nenyaviridae* sharing the same host, with *Vilyaviridae* infecting a separate one. Further, they provide a practical framework to identify virus–host clusters in an unbiased way with no a priori knowledge of the potential host required.

### Virus families occur alongside specific host genera in human stool

At the outset of investigation, we focused on the association between CRESS viruses and both *Entamoeba* and *Giardia* parasites collectively; however, evidence from endogenous viral elements and patterns of recombination among discovered viruses suggested that *Naryaviridae* and *Nenyaviridae* infect *Entamoeba*, while *Vilyaviridae* infect *Giardia*. We, therefore, tested the statistical associations of the families to their specific proposed host in human samples using Pearson’s chi-squared test, grouping *Naryaviridae* and *Nenyaviridae* together because of recombination between their genomes. Across all 374 study subjects, *Naryaviridae* and *Nenyaviridae* were strongly associated with *Entamoeba* parasites (*χ*^2^ = 32.34, *p* < 0.001), but not with *Giardia* (*χ*^2^ = 0.57, *p* = 0.45), while *Vilyaviridae* were strongly associated with *Giardia* (*χ*^2^ = 99.8, *p* < 0.001). *Vilyaviridae* were also positively associated to *Entamoeba*, however at a greatly reduced significance compared to *Giardia* (*χ*^2^ = 5.17, *p* = 0.02). This result is likely explained by *Entamoeba* coinfections in all 3 *Vilyaviridae* positive samples; indeed, *Entamoeba* coinfection was found in 73% of all *Giardia* positive samples (Table 1 and Supplementary Table 2). Although the cohorts examined here may not be representative of wider parasite populations, the prevalence of *Nenyaviridae* or *Naryaviridae* virus infections was 13% among *Entamoeba* cases (18 of 138), while *Vilyaviridae* had a prevalence of 27% among *Giardia* cases (3 of 11). The observed association between the viruses and their hosts in stool enabled a preliminary investigation into the biogeographic distribution of the three families. We mapped reads from public metagenome datasets derived from faecally polluted wastewater or primate stool to our viral genomes. We found reads from *Naryaviridae*, *Nenyaviridae*, and *Vilyaviridae* were detectable in the datasets examined, sourced from localities across North and South America, Europe, Africa, and Asia (Supplementary Fig. 4). This suggests the virus distributions are large, mirroring those of the hosts.

## Discussion

Here we report three CRESS virus families, *Naryaviridae* and *Nenyaviridae* infecting *Entamoeba*, and *Vilyaviridae* infecting *Giardia duodenalis*. Our study expands the number of CRESS families known to infect eukaryotes from five to eight, including the only groups recognised to infect protozoa. The investigation provides the only genome sequences of viruses infecting *Entamoeba*, nearly 50 years after the first of a series of papers studying infectious agents causing cell lysis in axenic *E. histolytica* culture^37^. For *Giardia*, one RNA virus species in the *Totiviridae* (Giardia lamblia virus) was discovered in 1986^38^, and the *Vilyaviridae* represent the second group of viruses. The discovery of viruses infecting *Entamoeba* and *Giardia*—collectively responsible for 300 million human disease cases annually^39^—should precipitate investigation of their potential impact on the clinical outcome of parasite infection. It is understood that only a subset of *Entamoeba* and *Giardia* infections result in symptomatic disease^40,41^, however, not all the factors underlying case variation are resolved. For example, *E. histolytica* interactions with gut bacteria are thought to play a role in pathogenesis^42^, but the effects of viruses are unexplored. As viruses can modulate parasite pathogenicity directly or indirectly via interaction with human immunity, they may result in parasite hypovirulence^11^ or hypervirulence^43^.

A large proportion of recognised virus genomes are divorced from their biological hosts. Targeted virus discovery from potential host taxa has a vital role to play in resolving this^44^, however, in instances of hosts intractable to culture, high-throughput methods must rely on viral genome sequences alone. Machine-learning algorithms trained on viral sequences with known hosts offer one possible approach^45^; however, due to their reliance on conserved sequence signals between training and test data, they will suffer from increasingly coarse prediction for divergent viruses. As we show, construction of viral recombination networks provides direct and unbiased biological evidence of shared hosts among virus genomes, even when individual genes are highly divergent or non-homologous. Given the highly consequential roles protozoa play in global health and ecosystem processes, deciphering additional unknown virus–host relationships among them is imperative.

## Methods

### Clinical samples

The 374 human subjects analysed here were from two cohorts. Cohort 1: stool samples of 194 HIV-1 infected individuals not on active antiretroviral therapy, who visited the out-patient clinic at the Amsterdam Medical Center in 1994 and 1995, as part of a study on unexplained diarrhoea^46,47^. Criteria for inclusion in the study were proven HIV-1 infection and being aged 18 years or older. Cohort 2: Stool samples of 85 HIV-1 positive and 95 HIV-1 negative men having sex with men (MSM) as part of the ACS, a prospective cohort study among HIV-positive and HIV-negative MSM, initiated in 1984^48^. Studies were approved by the Medical Ethics Committee of the Amsterdam University Medical Center, the Netherlands (MEC 07/182). Written informed consent of each participant was obtained at enrolment of both cohorts.

### VIDISCA library preparation and sequencing of human faecal samples

At collection, faecal samples were suspended 1:3 in broth containing penicillin, streptomycin, and amphotericin B, and stored at −80 °C until processing. Sample suspension (150 µl) was transferred to a reaction tube and centrifuged (10 min at 5000 g) to pellet solid matter and cellular debris. Supernatant was treated with 20 µl TURBO DNase (Thermo Fisher Scientific, Waltham, MA, USA) for 30 min at 37 °C (to remove naked DNA). Nucleic acids were extracted using the Boom method^49^ and reverse transcription was done using non-ribosomal hexamer primers designed to avoid mammal rRNA sequences^50^. This was followed by second strand synthesis and a cleanup via phenol/chloroform extraction and ethanol precipitation. Library preparation for the two cohorts varied from this point, since two different sequencing technologies were used. For cohort 1 standard VIDISCA library preparation was carried out^51^. Briefly, double-stranded DNA was digested with Mse1 restriction enzyme, and sequencing adapters were ligated to sticky ends. Libraries were amplified before size selection of fragments between 200 and 600 bp, quantification, and pooling. Sequencing was then done on an IonTorrent PGM instrument. For cohort 2, double-stranded DNA was fragmented to an average length of 400–500 bp, sequencing adapters were ligated, and libraries were amplified before sequencing with Illumina MiSeq instruments (150 bp paired end)^52^. Sequence reads associated with this study have been deposited in the European Nucleotide Archive (ENA) under study accession PRJEB35571.

### CRESS virus identification and characterisation

Sequence reads from cohort 1 were analysed to discover viruses^53^. Briefly, non-rRNA reads were identified using SortMeRNA v2.1^54^ and made non-redundant using CD-HIT v4.7^55^. Non-redundant reads were then aligned to viral proteins using UBLAST^56^, and false positives were reduced via BLASTn^57^ alignment of putative viral matches to the GenBank non-redundant nucleotides. Outputs were visualised with KronaTools v2.7^58^ and inspected to identify candidate CRESS virus reads. Two genomes were amplified via inverse PCR, the sequences of which were determined using Sanger sequencing (accessions MT293412.1 and MT293415.1). All primers are reported in Supplementary Table 10. An iterative search procedure was then carried out to identify additional samples containing related CRESS viruses. Predicted protein sequences were extracted from the two genomes and used as queries against reads from cohort 1 using UBLAST. This was also carried out against contigs assembled from cohort 2 sequencing data using SPAdes v3.5.0^59^. Further putative CRESS virus hits were manually curated or completed with Sanger sequencing, and were then used in subsequent searches. The process resulted in a final count of 20 CRESS virus coding sequences, 18 of which were complete genomes.

To determine a final list of samples regarded as virus positive, sequence reads from each cohort were mapped to the 20 virus coding sequences using BWA-MEM v0.7.17^60^. Reads mapping to multiple references were reassigned to their single most-likely reference using the PathoID module of PathoScope v2.0.7^61^. High-depth Illumina sequencing is prone to barcode swapping within flow cells, which may result in false positives; therefore, for cohort 2 a cutoff was imposed for a sample to be regarded as positive. Specifically, virus reads from *Entamoeba*-infecting or *Giardia*-infecting families had to make up at least 0.05% of sample reads (in instances where samples had received repeat sequencing, only the run receiving the highest number of sequences was analysed). In addition to the sequencing-based approach described, any PCR positive samples were also included.

Virus protein sequences extracted from open reading frames were queried against the Reference Proteome database with pHMMER^62^ and best hits were recorded. DNA secondary structure surrounding the putative nonanucleotide origin motif was assessed using MFOLD^63^ to confirm it was situated on a predicted stem loop. Circularity of viruses was confirmed by visual inspection of genomes and mapped reads, specifically reads that overlapped with both the beginning and end of genome sequences. To confirm that viral DNA was protected by a capsid, supernatant was first passed through a filter with 1200 nm pores, then 200 nm (GE Healthcare Life Sciences, Chicago, USA), followed by treatment with TURBO DNase (Thermo Fisher Scientific, Waltham, MA, USA). Subsequently viral nucleic acid was extracted with the Boom method, and PCR was carried out. To compare CRESS virus GC-content with that of their hosts, the Virus–Host DB^64^ was used in conjunction with the GenBank genomes resource to compile this information for virus–host pairs.

### Parasitological typing

Faecal samples from cohort 1 were examined by light microscopy for the presence of intestinal parasites (with both direct smears and concentrations using the Ridley technique). From both cohorts, sequence reads were mapped using BWA-MEM to parasite ribosomal RNA reference sequences, with aligning sequences then queried against the GenBank non-redundant nucleotide database. Reads with the best hit to a parasite ribosomal RNA reference, and a minimum alignment of 50 nt at over 95% nucleotide identity was retained as hits. Hits were aligned to diagnostic parasite reference sequences to type the parasite species where possible. Sequence reads were also mapped using BWA-MEM to predicted mRNA sequences from parasite genomes, specifically *E. histolytica* (GCF_000208925.1) and *G. duodenalis* (GCF_000002435.1). Predicted mRNA databases were first curated using identity searches to remove sequences derived from endogenous viral elements and ribosomal RNA. Hits were also filtered to allow only those with a minimum alignment of 50 nt at over 95% nucleotide identity to their respective subject sequence. The possibility of barcode swapping in cohort 2 Illumina data led us to impose a cutoff for a sample to be called as positive; specifically, the parasite sequence reads as a percentage of the total reads had to be greater than the lower quartile value. For a selection of samples from cohort 1, confirmatory testing was done with *E. histolytica* and *E. dispar* diagnostic qPCRs, in addition to *Entamoeba* generic PCR combined with Sanger sequencing of amplicons. Due to generally low read counts observed for *Giardia*, all 21 virus-positive samples were subjected to a confirmatory *Giardia* diagnostic qPCR. The prevalence of *Giardia* infection among our cohort participants was 2.94% (11 of 374), and the prevalence of *Entamoeba* infection was 36.90% (138 of 374). Our participants were 93% MSM, and these *Giardia* and *Entamoeba* frequencies are concordant with previously reported data from this demographic (from 1% to 18% for *Giardia* with a median of 5% infection, and from 3% to 33% for *Entamoeba* with a median of 22% infection^65^). To confirm that other protozoa were not the viral hosts, the 21 virus-positive samples were tested for additional parasites: *Dientamoeba*, *Cryptosporidium*, and *Blastocystis* were tested by diagnostic qPCR, while *Endolimax, Chilomastix*, *Pentatrichomonas*, and *Retortamonas* 18S rRNA sequences were analysed in the same manner described above.

### Endogenous viral element analysis

CRESS virus genomes were aligned to GenBank databases: the non-redundant nucleotide using BLASTn, the non-redundant protein using BLASTx, and the whole-genome shotgun contigs of *Entamoeba* and *Giardia* using BLASTn. Nucleotide and protein sequences of hits were extracted and manually curated to use in subsequent analyses. Comparison between independent assemblies of *E. histolytica* and *G. duodenalis* (to confirm consistency of endogenous viral element presence) was done using BLASTn of endogenous *Rep* gene elements from each genus against each assembly, recording the best aligning hit. Pairwise comparisons between sequences were all performed using BLAT via the MAFFT online server^66^. Available genome assemblies from relatives of *Entamoeba* and *G. duodenalis* were also analysed for the presence of elements, specifically *Mastigamoeba balamuthi* (GCA_902651635.1), *Spironucleus salmonicida* (GCA_000497125.1), *Trichomonas vaginalis* (GCA_000002825.1), and *G. muris* (GCA_006247105.1); however, none of these assemblies contained elements belonging to the *Naryaviridae*, *Nenyaviridae*, or *Vilyaviridae*. To assess read coverage across *E. histolytica* contig NW_001915013.1, raw sequencing reads were downloaded from the TraceDB (isolate HM1:IMSS, https://ftp.ncbi.nlm.nih.gov/pub/TraceDB/entamoeba_histolytica/) and ENA (isolate KU27, accessions SRR071802 and SRR072203). BWA-MEM was used to map reads to the complete reference contig, followed by visualisation of coverage using CodonCode Aligner v9.0.1. Easyfig v2.2.5^67^ was used to visualise pairwise identity between *G. duodenalis* contigs VSRU01000012.1 and AHHH01000265.1. To identify evidence of an RNA interference response against endogenous viral elements, BWA-backtrack^60^ was used to map *E. histolytica* AGO2-2 associated small RNAs from ENA project PRJNA187070^32^ against contigs containing elements from the *E. histolytica* RefSeq genome assembly (GCA_000208925.2). Prior to mapping, sequencing adapters were trimmed using BBDuk (http://jgi.doe.gov/data-and-tools/bb-tools/), and sequences over 40 nt and under 15 nt were discarded. Reads mapping with zero sequence mismatches were retained, and coverage of contigs was calculated using the SAMtools mpileup utility^68^. Positions of endogenous viral elements and small RNA coverages were visualised for a selection of contigs using Circos v0.69-8^69^.

### Phylogenetic analysis and pairwise protein comparison

Phylogenetic analysis of the Rep protein utilised a previously compiled chimaera-free dataset^13^, with the addition of the *Redondoviridae*^18^, five viral sequences found during BLASTn searches of the GenBank non-redundant nucleotide database, and our CRESS virus sequences (both exogenous and endogenous viruses). Rep proteins were aligned using MAFFT v7^66^ with the L-INS-i option leaving gappy regions unaligned. The resulting alignment was trimmed using trimAl v1.4^70^ set to gappyout. Maximum-likelihood phylogenetic analysis was performed using RaxML v8.2.9^71^ with the PROTCATGTR substitution model and automatic bootstopping, which stopped rapid bootstrap searching after 350 replicates. Treefiles were visualised using Figtree v1.4.4 (https://github.com/rambaut/figtree/releases). The same methods were applied for phylogenetic analysis of the three Rep protein families in isolation, as well as their corresponding Cap proteins. For delimitation of Rep genera, pairwise comparison was carried out using the online tool SIAS (available at: http://imed.med.ucm.es/Tools/sias.html), with the denominator set to mean length of sequences.

### Public metagenome data

Data to estimate the global distribution of parasite-infecting CRESS viruses was obtained from a number of public metagenomes and mapped using BWA-MEM to virus genomes. Wastewater samples from ENA project PRJNA169010^72^ were from Maiduguri (Nigeria), Kathmandu (Nepal), Bangkok (Thailand), and San Francisco (USA); project PRJNA70623^73^ samples were from Addis Ababa (Ethiopia), Barcelona (Spain), and Pittsburgh (USA); project PRJNA322301^74^ was from Tallahassee (USA); project PRJNA434744^75^ was from Cincinnati (USA); and project PRJNA385831^76^ was from Sheboygan (USA). Macaque stool from project PRJNA299332^14^ was from the California National Primate Research Center (USA). Human stool from project PRJNA418044^26^ was from Caracas (Venezuela) and remote villages in South-East Venezuela; and project PRJEB9524^77^ was from Uganda. A further site was annotated based on a public virus genome (LC406405.1) which clustered within the *Vilyaviridae*, sampled from a cat in Japan^78^.

### Reporting summary

Further information on research design is available in the Nature Research Reporting Summary linked to this article.

## Supplementary information

Supplementary Information

Reporting Summary

## Data Availability

Viral genomes and coding sequences are available under NCBI accessions MT293410.1–MT293429.1. Raw sequencing reads are available under European Nucleotide Archive study accession PRJEB35571. Protein alignments and tree files are available from Figshare (https://figshare.com/projects/Entamoeba_and_Giardia_parasites_implicated_as_hosts_of_CRESS_viruses/84065). GenBank databases are available via NCBI (https://www.ncbi.nlm.nih.gov/), and the Reference Proteome database was integrated with the pHMMER web service (https://www.ebi.ac.uk/Tools/hmmer/search/phmmer).
