# Supplementary material for: Entamoeba and Giardia parasites implicated as hosts of CRESS viruses
Source: Nat Commun. 2020 Sep 15;11:4620. doi: 10.1038/s41467-020-18474-w (PMC7493932; doi:10.1038/s41467-020-18474-w)
Supplement: Supplementary file 1 — Supplementary Information [file 41467_2020_18474_MOESM1_ESM.pdf]

Supplementary Information for manuscript:

Entamoeba and *Giardia* parasites implicated as hosts of CRESS viruses

Kinsella *et al*.

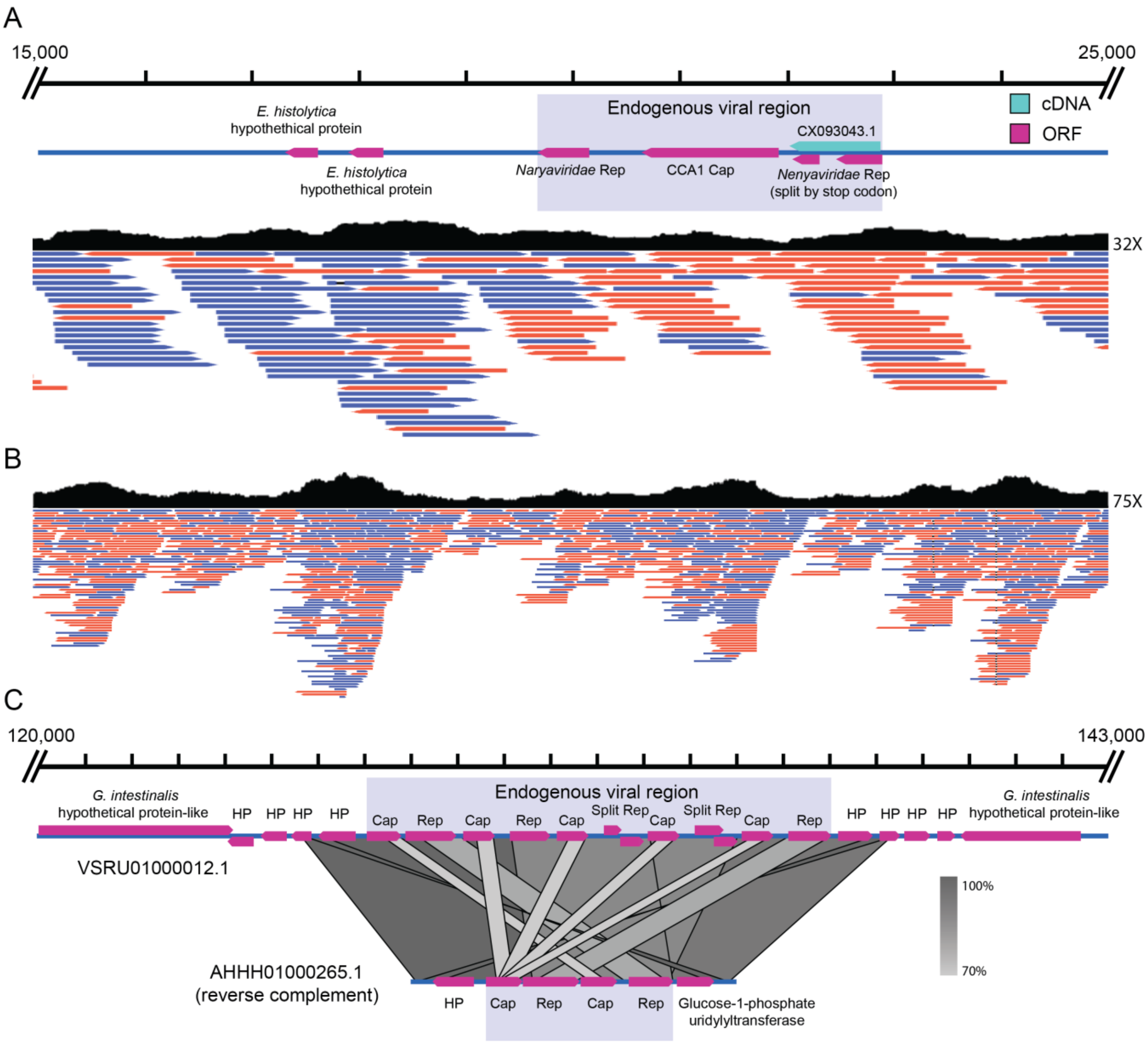

Supplementary Figure 1. The presence of endogenous viral elements is supported by raw-read coverage or long-reads. (A) Sanger sequencing reads from *Entamoeba histolytica* isolate HM1:IMSS (isolated in Mexico, 1967) aligned to genomic contig NW_001915013.1, revealing coverage spanning both the endogenous virus element junctions and flanking host sequence. Maximum coverage depth is shown to the right of the coverage plot. (B) Combined 454 and Illumina sequencing reads from *E. histolytica* isolate KU27 (isolated in Japan, 2001) aligned to contig NW_001915013.1, confirming the element is shared among independent isolates. (C) Long-read technology assists in resolution of repetitive genomic features, as shown by *Giardia duodenalis* genomic contig VSRU01000012.1, which contains a 10 kb region of tandemly repeated integrated viral genomes belonging to the *Vilyaviridae*. This contig belongs to a hybrid assembly built from nanopore long-reads and conventional short-reads[1] (GCA_011634595.1). Host hypothetical proteins are annotated as 'HP'. BLASTn alignment with the short-read based contig AHHH01000265.1 reveals that the two share both the viral integration and neighbouring host sequences; however the latter contig has a shorter endogenous viral region. This is likely a result of assembly software being unable to distinguish between tandem repeat units, leading to assembly collapse. Regions of BLASTn alignment are shown by the grey blocks, with shade corresponding to % identity.

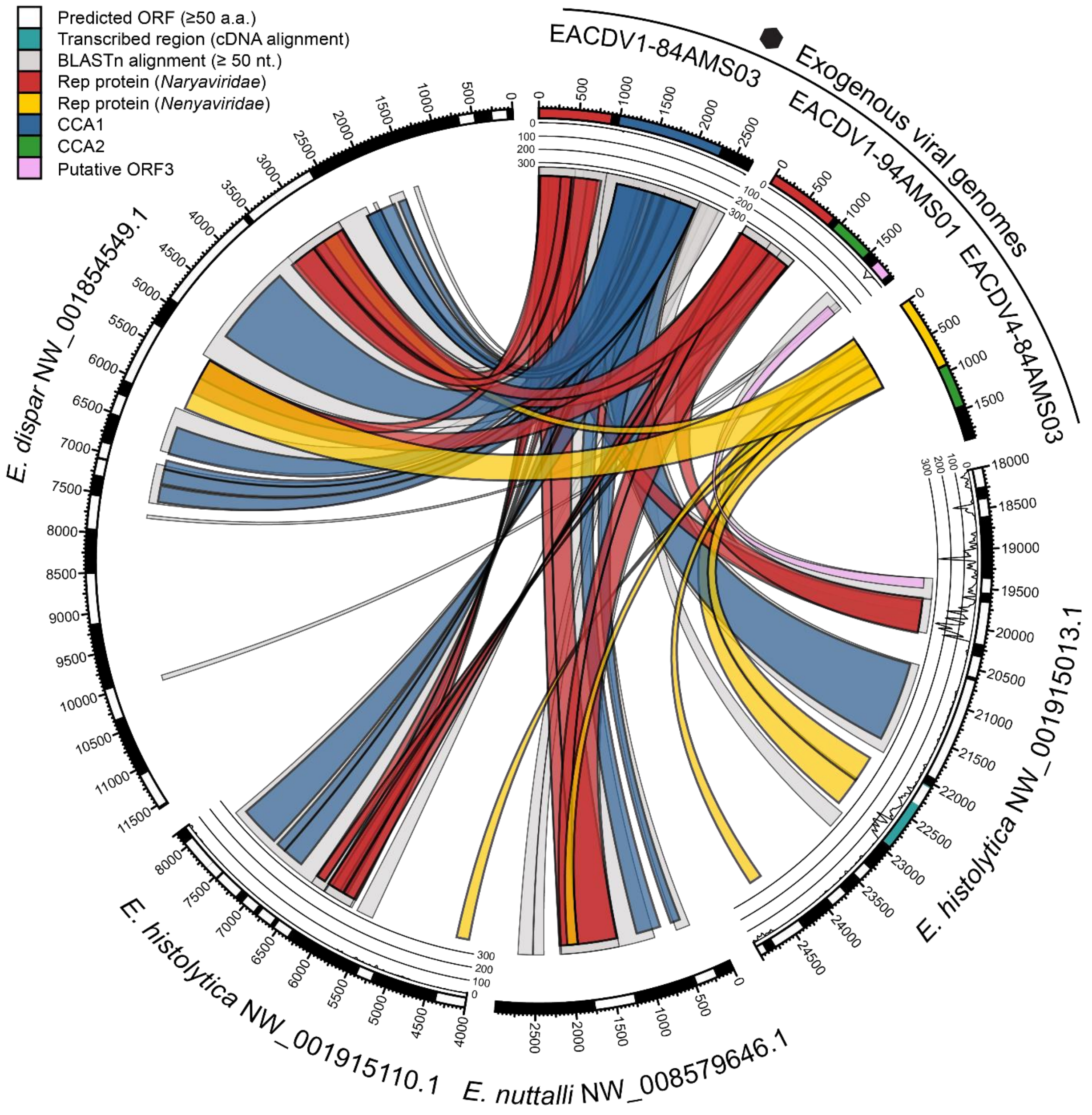

Supplementary Figure 2. Virus genome integrations within *Entamoeba* genomes, and subsequent small RNA control. Alignments were generated using BLASTn (grey bands) and tBLASTn (coloured bands) between three viral genomes (top right, EACDV1-84AMS03 = MT293413, EACDV1-94AMS01 = MT293412, and EACDV4-84AMS03 = MT293420) and contigs from three species of *Entamoeba*. Regions of alignment were visualised with Circos[2]. Open reading frames on parasite contigs were coloured white, while those of exogenous viruses were coloured according to the legend. Alignments reveal a whole viral genome integration (between nucleotides 2,600 and 5,300 of *E. dispar* contig NW_001854549.1), and also multiple unrelated integrations by *Naryaviridae* and *Nenyaviridae* in close physical proximity to each other. Notably, this latter observation suggests CRESS integration is sometimes site-specific, and this is most likely mediated by Rep recognition of previously integrated nonanucleotide origin motifs within the host genome, a model discussed elsewhere[3]. To add support that endogenous viral elements are real features of parasite genomes, rather than artefacts in genome assemblies, *E. histolytica* contigs are annotated with AGO2-2 associated small RNA coverage[4]. Data from other *Entamoeba* species was not available. Coverage peaks (inner ring) often occur across open reading frames derived from virus integrations, suggesting these are real genomic features. Some small RNAs also exactly match exogenous viral genomes, implying a possible indirect role in antiviral defence. CCA = CRESS virus *Cap* assemblage.

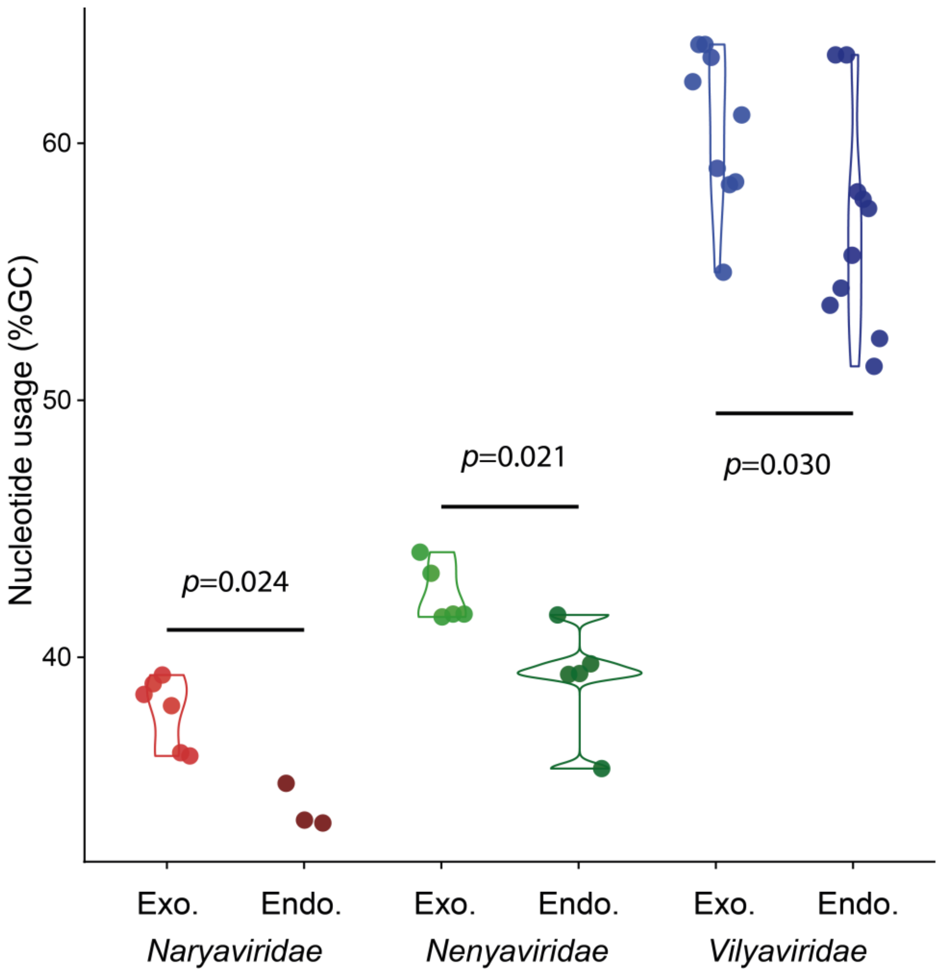

Supplementary Figure 3. GC-content of exogenous and endogenous viruses. Points denote GC-content of a viral *Rep* gene sequence, either from the genome of an exogenous virus (Exo.) or extracted from an endogenous viral element within a host genome (Endo.). For each viral family, Mann-Whitney U tests were performed to evaluate whether exogenous and endogenous sequences had significantly different GC-content than expected by chance (*Naryaviridae* n=9, *Nenyaviridae* n=10, *Vilyaviridae* n=19). In each case GC-content was found to be different; exogenous virus genomes have a significantly higher GC-content than related endogenous viral elements.

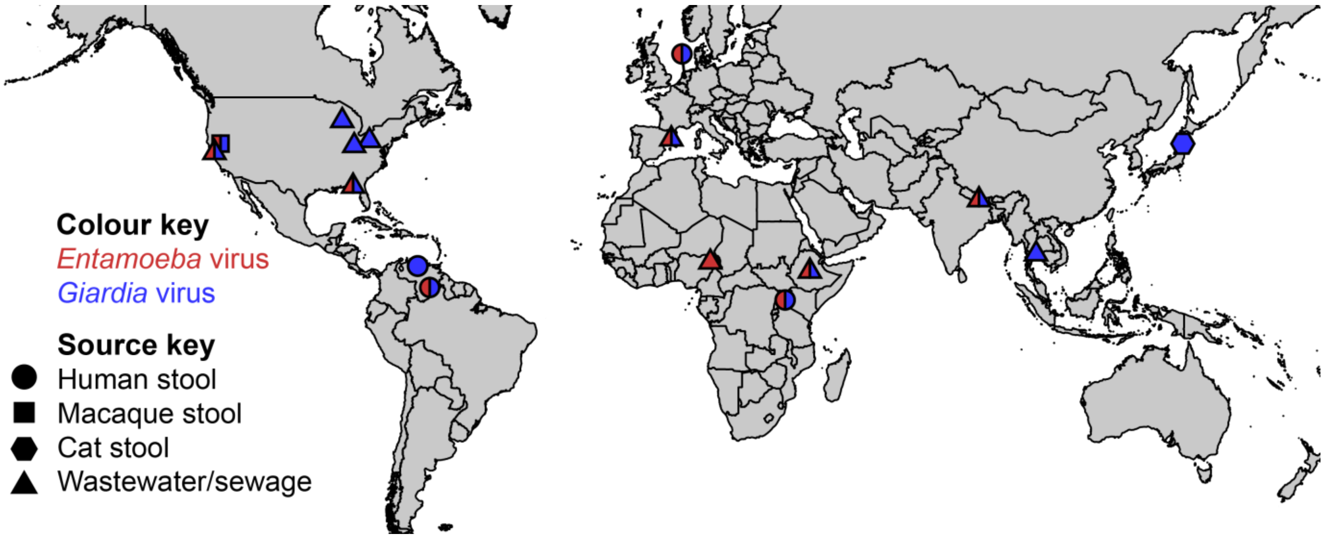

Supplementary Figure 4. Geographical origin of public viral genomes or metagenome samples containing parasite-infecting CRESS viruses. Shape of symbols denotes the sample source type, while colour denotes whether the identified viruses were *Entamoeba*-infecting (*Naryaviridae* and *Nenyaviridae*), *Giardia*-infecting (*Vilyaviridae*), or both (bicolour symbols). The map was adapted from world border templates available from http://thematicmapping.org/downloads/world_borders.php, provided by Bjørn Sandvik under a Creative Commons Attribution-ShareAlike 3.0 license (https://creativecommons.org/licenses/by-sa/3.0/legalcode).

Supplementary Table 1. Genomic features of parasite infecting CRESS viruses. Origin motifs[5] were identified, and the conserved stem loop was confirmed using MFOLD[6]. Viral proteins were queried using pHMMER[7] against reference proteomes to find their closest relatives, with only top hits reported. All putative Rep and Cap proteins had significant similarity to representatives of these genes, however sometimes this was in a secondary hit and was therefore not reported below. E-value is the expected likelihood of the given alignment occurring by chance.

				Replication-associated protein			Capsid protein		
**Virus species, isolate**	**Accession**	**Family**	**Origin motif**	**Best hit name**	**Source species**	**E-value**	**Best hit name**	**Source species**	**E-value**
Entamoeba-associated CRESS DNA virus 1, 84-AMS-01	MT293410	Naryaviridae	TAGTATTAC	Uncharacterized protein	Entamoeba histolytica	1.50E-78	Uncharacterized protein (Fragment)	Punica granatum	2.60E-26
Entamoeba-associated CRESS DNA virus 1, 84-AMS-02	MT293411	Naryaviridae	TAGTATTAC	Uncharacterized protein	Entamoeba histolytica	7.60E-80	Uncharacterized protein (Fragment)	Punica granatum	2.60E-26
Entamoeba-associated CRESS DNA virus 1, 94-AMS-01	MT293412	Naryaviridae	TAGTATTAC	Uncharacterized protein	Entamoeba histolytica	7.60E-80	Uncharacterized protein (Fragment)	Punica granatum	2.70E-29
Entamoeba-associated CRESS DNA virus 1, 84-AMS-03	MT293413	Naryaviridae	TAGTATTAC	Uncharacterized protein	Entamoeba histolytica	3.40E-77	Uncharacterized protein	Entamoeba histolytica	1.00E-147
Entamoeba-associated CRESS DNA virus 2, 84-AMS-01	MT293414	Naryaviridae	TAGTATTAC	Uncharacterized protein	Entamoeba histolytica	3.10E-87	Putative capsid protein	Duck faeces associated circular DNA virus 3	6.60E-11
Entamoeba-associated CRESS DNA virus 2, 94-AMS-01	MT293415	Naryaviridae	TAGTATTAC	Uncharacterized protein	Entamoeba histolytica	2.20E-87	Putative capsid protein	Duck faeces associated circular DNA virus 3	2.80E-13
Entamoeba-associated CRESS DNA virus 3, 84-AMS-01	MT293416	Nenyaviridae	TAATATTAC	Replication-associated protein	Odonata-associated circular virus-14	1.30E-72	Uncharacterized protein	Entamoeba histolytica	2.80E-44
Entamoeba-associated CRESS DNA virus 3, 84-AMS-02	MT293417	Nenyaviridae	TAATATTAC	Replication-associated protein	Odonata-associated circular virus-14	4.90E-71	Uncharacterized protein	Entamoeba histolytica	2.70E-44
Entamoeba-associated CRESS DNA virus 4, 84-AMS-01	MT293418	Nenyaviridae	AAGTATTAC	Replication-associated protein	Odonata-associated circular virus-14	2.30E-54	Putative capsid protein	Duck faeces associated circular DNA virus 3	6.30E-12
Entamoeba-associated CRESS DNA virus 4, 84-AMS-02	MT293419	Nenyaviridae	AAATATTAC	Replication-associated protein	Odonata-associated circular virus-14	4.60E-55	Putative capsid protein	Duck faeces associated circular DNA virus 3	2.80E-11
Entamoeba-associated CRESS DNA virus 4, 84-AMS-03	MT293420	Nenyaviridae	AAATATTAC	Replication-associated protein	Odonata-associated circular virus-14	4.60E-55	Putative capsid protein	Duck faeces associated circular DNA virus 3	1.90E-12
Giardia-associated CRESS DNA virus 1, 84-AMS-01	MT293421	Vilyaviridae	TAGTATTAC	Replication-associated protein	Fly associated circular virus 7	1.60E-129	Putative capsid protein	Feline stool-associated circular virus KU14	4.50E-42
Giardia-associated CRESS DNA virus 1, 84-AMS-02	MT293422	Vilyaviridae	TAGTATTAC	Replication-associated protein	Fly associated circular virus 7	1.60E-129	Putative capsid protein	Feline stool-associated circular virus KU14	2.30E-42
Giardia-associated CRESS DNA virus 1, 84-AMS-03	MT293423	Vilyaviridae	TAGTATTAC	Replication-associated protein	Fly associated circular virus 7	2.50E-130	Putative capsid protein	Feline stool-associated circular virus KU14	2.70E-43
Giardia-associated CRESS DNA virus 1 CDS, 84-AMS-04	MT293424	Vilyaviridae	NA	Replication-associated protein	Fly associated circular virus 7	1.90E-131	Putative capsid protein	Feline stool-associated circular virus KU14	5.40E-45
Giardia-associated CRESS DNA virus 2, 84-AMS-01	MT293425	Vilyaviridae	TAGTATTAC	Replication-associated protein	Fly associated circular virus 7	1.60E-90	Putative capsid protein	Feline stool-associated circular virus KU14	1.60E-75
Giardia-associated CRESS DNA virus 2, 84-AMS-02	MT293426	Vilyaviridae	CAGTATTAC	Replication-associated protein	Fly associated circular virus 7	1.60E-87	Putative capsid protein	Feline stool-associated circular virus KU14	1.10E-71
Giardia-associated CRESS DNA virus 3 CDS, 84-AMS-01	MT293427	Vilyaviridae	NA	Putative replication associated protein	Feline stool-associated circular virus KU14	7.00E-136	Putative capsid protein	Feline stool-associated circular virus KU14	3.00E-47
Giardia-associated CRESS DNA virus 3, 84-AMS-02	MT293428	Vilyaviridae	TAGTATTAC	Putative replication associated protein	Feline stool-associated circular virus KU14	6.90E-136	Putative capsid protein	Feline stool-associated circular virus KU14	7.90E-48
Giardia-associated CRESS DNA virus 4, 84-AMS-01	MT293429	Vilyaviridae	TAGTATTAC	Putative replication associated protein	Feline stool-associated circular virus KU14	9.00E-154	Putative capsid protein	Feline stool-associated circular virus KU14	1.10E-74

Supplementary Table 2. Samples positive for *Entamoeba*-infecting or *Giardia*-infecting CRESS viruses. The cohort of each positive sample is shown, as well as the identified parasite species, or genus where species level determination was not possible.

Number	Cohort	Sample	Parasite	Virus family	Additional detected protozoa
1	1	94_F005	*Entamoeba coli*	*Naryaviridae*	*Blastocystis hominis*<br>*Endolimax nana*
2	1	94_F011	*Entamoeba dispar*	*Naryaviridae*	
3	1	94_F038	*Entamoeba dispar*	*Naryaviridae*	
4	1	94_F095	*Entamoeba hartmanni*	*Naryaviridae*<br>*Nenyaviridae*	
5	1	94_F211	*Entamoeba dispar*	*Naryaviridae*	
6	2	84_F022	*Entamoeba hartmanni*	*Nenyaviridae*	
7	2	84_F033	*Entamoeba dispar*	*Naryaviridae*	
8	2	84_F059	*Entamoeba dispar*	*Naryaviridae*	*Endolimax nana*
9	2	84_F068	*Entamoeba dispar*	*Naryaviridae*	*Blastocystis hominis*
10	2	84_F069	*Entamoeba dispar*	*Nenyaviridae*	*Endolimax nana*
11	2	84_F070	*Entamoeba coli*<br>*Entamoeba hartmanni*	*Naryaviridae*	*Endolimax nana*
12	2	84_F079	*Entamoeba hartmanni*	*Naryaviridae*<br>*Nenyaviridae*	*Blastocystis hominis*<br>*Endolimax nana*
13	2	84_F098	*Entamoeba*	*Nenyaviridae*	
14	2	84_F117	*Entamoeba hartmanni*	*Naryaviridae*<br>*Nenyaviridae*	
15	2	84_F130	*Giardia duodenalis*<br>*Entamoeba coli*	*Vilyaviridae*	*Endolimax nana*
16	2	84_F142	*Entamoeba hartmanni*	*Nenyaviridae*	*Endolimax nana*
17	2	84_F165	*Entamoeba*	*Naryaviridae*	*Endolimax nana*
18	2	84_F175	*Entamoeba hartmanni*	*Naryaviridae*<br>*Nenyaviridae*	*Endolimax nana*
19	2	84_F186	*Giardia duodenalis*<br>*Entamoeba coli*<br>*Entamoeba hartmanni*	*Vilyaviridae*	*Endolimax nana*
20	2	84_F188	*Entamoeba*	*Naryaviridae*<br>*Nenyaviridae*	
21	2	84_F198	*Giardia duodenalis*<br>*Entamoeba hartmanni*	*Vilyaviridae*	

Supplementary Table 3. Viruses are related to both virus and parasite nucleotide sequences. Top alignments of viral genomes and coding sequences are shown after BLASTn alignment to the NCBI non-redundant nucleotide database. Among top hits, several viruses aligned to both viral and parasite derived sequences.

Virus species, isolate	Accession	Family	Top hit	Accession	Query coverage (%)	Identity (%)	E-value
Entamoeba-associated CRESS DNA virus 1, 84-AMS-01	MT293410	Naryaviridae	Entamoeba nuttalli P19 hypothetical protein partial mRNA	XM_008862058.1	22	78	2.00E-106
Entamoeba-associated CRESS DNA virus 1, 84-AMS-02	MT293411	Naryaviridae	Entamoeba nuttalli P19 hypothetical protein partial mRNA	XM_008862058.1	23	80	5.00E-108
Entamoeba-associated CRESS DNA virus 1, 94-AMS-01	MT293412	Naryaviridae	Entamoeba nuttalli P19 hypothetical protein partial mRNA	XM_008862058.1	23	79	1.00E-102
Entamoeba-associated CRESS DNA virus 1, 84-AMS-03	MT293413	Naryaviridae	Entamoeba dispar SAW760 hypothetical protein EDI_137180 mRNA, complete cds	XM_001738173.1	18	97	0
Entamoeba-associated CRESS DNA virus 2, 84-AMS-01	MT293414	Naryaviridae	Unidentified circular ssDNA virus isolate cg6319, complete genome	KU043415.1	31	73	7.00E-81
Entamoeba-associated CRESS DNA virus 2, 94-AMS-01	MT293415	Naryaviridae	Unidentified circular ssDNA virus isolate cg6319, complete genome	KU043415.1	31	69	1.00E-46
Entamoeba-associated CRESS DNA virus 3, 84-AMS-01	MT293416	Nenyaviridae	Unidentified circular ssDNA virus clone V16C Rep and hypothetical protein genes, complete cds	MG571899.1	86	89	0
Entamoeba-associated CRESS DNA virus 3, 84-AMS-02	MT293417	Nenyaviridae	Unidentified circular ssDNA virus clone V16C Rep and hypothetical protein genes, complete cds	MG571899.1	86	90	0
Entamoeba-associated CRESS DNA virus 4, 84-AMS-01	MT293418	Nenyaviridae	Circoviridae sp. isolate ctga69, complete genome	MH617639.1	53	78	0
Entamoeba-associated CRESS DNA virus 4, 84-AMS-02	MT293419	Nenyaviridae	Circoviridae sp. isolate ctga69, complete genome	MH617639.1	53	78	0
Entamoeba-associated CRESS DNA virus 4, 84-AMS-03	MT293420	Nenyaviridae	Circoviridae sp. isolate ctga69, complete genome	MH617639.1	53	78	0
Giardia-associated CRESS DNA virus 1, 84-AMS-01	MT293421	Vilyaviridae	Giardia intestinalis putative replication-accociated protein REP1 (REP1) and putative replication-associated protein REP2 (REP2) genes, complete cds; and unknown gene	AF059664.1	94	73	0
Giardia-associated CRESS DNA virus 1, 84-AMS-02	MT293422	Vilyaviridae	Giardia intestinalis putative replication-accociated protein REP1 (REP1) and putative replication-associated protein REP2 (REP2) genes, complete cds; and unknown gene	AF059664.1	94	73	0
Giardia-associated CRESS DNA virus 1, 84-AMS-03	MT293423	Vilyaviridae	Giardia intestinalis putative replication-accociated protein REP1 (REP1) and putative replication-associated protein REP2 (REP2) genes, complete cds; and unknown gene	AF059664.1	94	73	0
Giardia-associated CRESS DNA virus 1 CDS, 84-AMS-04	MT293424	Vilyaviridae	Giardia intestinalis putative replication-accociated protein REP1 (REP1) and putative replication-associated protein REP2 (REP2) genes, complete cds; and unknown gene	AF059664.1	94	68	1.00E-179
Giardia-associated CRESS DNA virus 2, 84-AMS-01	MT293425	Vilyaviridae	Giardia intestinalis putative replication-accociated protein REP1 (REP1) and putative replication-associated protein REP2 (REP2) genes, complete cds; and unknown gene	AF059664.1	57	81	3.00E-167
Giardia-associated CRESS DNA virus 2, 84-AMS-02	MT293426	Vilyaviridae	Virus NG10 replication-association protein (rep) gene, partial cds	GQ404895.1	31	92	0
Giardia-associated CRESS DNA virus 3 CDS, 84-AMS-01	MT293427	Vilyaviridae	Uncultured virus clone CG97, complete genome	KY487991.1	88	83	0
Giardia-associated CRESS DNA virus 3, 84-AMS-02	MT293428	Vilyaviridae	Uncultured virus clone CG97, complete genome	KY487991.1	89	95	0
Giardia-associated CRESS DNA virus 4, 84-AMS-01	MT293429	Vilyaviridae	Feline stool-associated circular virus KU7 DNA, complete genome	LC406405.1	60	72	2.00E-126

Supplementary Table 4. Viruses are related to both virus and parasite proteins. Top alignments of viral genomes and coding sequences are shown after BLASTx alignment to the NCBI non-redundant protein database. Among top hits, viruses aligned to both viral and parasite derived sequences.

Virus species, isolate	Accession	Family	Top hit	Accession	Query coverage (%)	Identity (%)	E-value
Entamoeba-associated CRESS DNA virus 1, 84-AMS-01	MT293410	Naryaviridae	hypothetical protein CL6EHI_168860 [Entamoeba histolytica]	GAT95322.1	41	48	2.00E-75
Entamoeba-associated CRESS DNA virus 1, 84-AMS-02	MT293411	Naryaviridae	hypothetical protein CL6EHI_168860 [Entamoeba histolytica]	GAT95322.1	42	48	2.00E-77
Entamoeba-associated CRESS DNA virus 1, 94-AMS-01	MT293412	Naryaviridae	hypothetical protein CL6EHI_168860 [Entamoeba histolytica]	GAT95322.1	42	48	2.00E-77
Entamoeba-associated CRESS DNA virus 1, 84-AMS-03	MT293413	Naryaviridae	hypothetical protein [Entamoeba dispar SAW760]	XP_001738226.1	43	63	2.00E-166
Entamoeba-associated CRESS DNA virus 2, 84-AMS-01	MT293414	Naryaviridae	Hypothetical protein EHI5A_163890 [Entamoeba histolytica KU27]	EMD43492.1	45	54	8.00E-90
Entamoeba-associated CRESS DNA virus 2, 94-AMS-01	MT293415	Naryaviridae	Hypothetical protein EHI5A_163890 [Entamoeba histolytica KU27]	EMD43492.1	45	54	5.00E-90
Entamoeba-associated CRESS DNA virus 3, 84-AMS-01	MT293416	Nenyaviridae	hypothetical protein [unidentified circular ssDNA virus]	AWU66058.1	43	86	0
Entamoeba-associated CRESS DNA virus 3, 84-AMS-02	MT293417	Nenyaviridae	hypothetical protein [unidentified circular ssDNA virus]	AWU66058.1	43	88	0
Entamoeba-associated CRESS DNA virus 4, 84-AMS-01	MT293418	Nenyaviridae	helicase [Circoviridae sp.]	AXH77589.1	47	85	0
Entamoeba-associated CRESS DNA virus 4, 84-AMS-02	MT293419	Nenyaviridae	helicase [Circoviridae sp.]	AXH77589.1	47	86	0
Entamoeba-associated CRESS DNA virus 4, 84-AMS-03	MT293420	Nenyaviridae	helicase [Circoviridae sp.]	AXH77589.1	48	86	0
Giardia-associated CRESS DNA virus 1, 84-AMS-01	MT293421	Vilyaviridae	RNA helicase [Giardia intestinalis]	ESU34896.1	41	73	2.00E-150
Giardia-associated CRESS DNA virus 1, 84-AMS-02	MT293422	Vilyaviridae	RNA helicase [Giardia intestinalis]	ESU34896.1	41	73	2.00E-150
Giardia-associated CRESS DNA virus 1, 84-AMS-03	MT293423	Vilyaviridae	RNA helicase [Giardia intestinalis]	ESU34896.1	41	73	3.00E-151
Giardia-associated CRESS DNA virus 1 CDS, 84-AMS-04	MT293424	Vilyaviridae	RNA helicase [Giardia intestinalis]	ESU34896.1	42	71	1.00E-138
Giardia-associated CRESS DNA virus 2, 84-AMS-01	MT293425	Vilyaviridae	helicase [CRESS virus sp.]	AYP28739.1	41	63	2.00E-126
Giardia-associated CRESS DNA virus 2, 84-AMS-02	MT293426	Vilyaviridae	replication-association protein [Virus NG10]	ADF80742.1	30	94	6.00E-138
Giardia-associated CRESS DNA virus 3 CDS, 84-AMS-01	MT293427	Vilyaviridae	Rep [uncultured virus]	AUM62049.1	45	97	0
Giardia-associated CRESS DNA virus 3, 84-AMS-02	MT293428	Vilyaviridae	Rep [uncultured virus]	AUM62049.1	44	97	0
Giardia-associated CRESS DNA virus 4, 84-AMS-01	MT293429	Vilyaviridae	putative replication associated protein [Feline stool-associated circular virus KU9]	BBF24945.1	41	75	1.00E-155

Supplementary Table 5. Whole and partial integrations of virus genomes in parasite genomes. Top alignments of viral genomes and coding sequences are shown after BLASTn alignment to parasite whole-genome shotgun contigs databases. This reveals several complete (i.e. high coverage) and partial whole genome integrations.

Virus species, isolate	Accession	Family	Top hit	Accession	Query coverage (%)	Identity (%)	E-value
Entamoeba-associated CRESS DNA virus 1, 84-AMS-01	MT293410	Naryaviridae	Entamoeba dispar SAW760 gcontig_1104316507336, whole genome shotgun sequence	AANV02000527.1	47	77.29	0
Entamoeba-associated CRESS DNA virus 1, 84-AMS-02	MT293411	Naryaviridae	Entamoeba dispar SAW760 gcontig_1104316507336, whole genome shotgun sequence	AANV02000527.1	47	77.59	0
Entamoeba-associated CRESS DNA virus 1, 94-AMS-01	MT293412	Naryaviridae	Entamoeba dispar SAW760 gcontig_1104316507336, whole genome shotgun sequence	AANV02000527.1	47	77.36	0
Entamoeba-associated CRESS DNA virus 1, 84-AMS-03	MT293413	Naryaviridae	Entamoeba dispar SAW760 gcontig_1104316507336, whole genome shotgun sequence	AANV02000527.1	100	84.25	0
Entamoeba-associated CRESS DNA virus 2, 84-AMS-01	MT293414	Naryaviridae	Entamoeba histolytica KU27 contig_1714, whole genome shotgun sequence	AOSC01001714.1	34	64.61	4.00E-17
Entamoeba-associated CRESS DNA virus 2, 94-AMS-01	MT293415	Naryaviridae	Entamoeba histolytica KU27 contig_1714, whole genome shotgun sequence	AOSC01001714.1	34	64.3	2.00E-14
Entamoeba-associated CRESS DNA virus 3, 84-AMS-01	MT293416	Nenyaviridae	Entamoeba invadens IP1 contig_3312, whole genome shotgun sequence	AANW03003312.1	2	88.33	1.00E-09
Entamoeba-associated CRESS DNA virus 3, 84-AMS-02	MT293417	Nenyaviridae	Entamoeba invadens IP1 contig_3312, whole genome shotgun sequence	AANW03003312.1	2	88.33	1.00E-09
Entamoeba-associated CRESS DNA virus 4, 84-AMS-01	MT293418	Nenyaviridae	NS	NS	NS	NS	NS
Entamoeba-associated CRESS DNA virus 4, 84-AMS-02	MT293419	Nenyaviridae	NS	NS	NS	NS	NS
Entamoeba-associated CRESS DNA virus 4, 84-AMS-03	MT293420	Nenyaviridae	NS	NS	NS	NS	NS
Giardia-associated CRESS DNA virus 1, 84-AMS-01	MT293421	Vilyaviridae	Giardia intestinalis strain DH DH_A2_0009, whole genome shotgun sequence	AHGT01000120.1	99	73.16	0
Giardia-associated CRESS DNA virus 1, 84-AMS-02	MT293422	Vilyaviridae	Giardia intestinalis assemblage A WGS project CAHQ00000000	CAHQ01000875.1	94	73.14	0
Giardia-associated CRESS DNA virus 1, 84-AMS-03	MT293423	Vilyaviridae	Giardia intestinalis strain DH DH_A2_0009, whole genome shotgun sequence	AHGT01000120.1	99	72.7	0
Giardia-associated CRESS DNA virus 1 CDS, 84-AMS-04	MT293424	Vilyaviridae	Giardia intestinalis assemblage A WGS project CAHQ00000000	CAHQ01000875.1	95	68.46	0
Giardia-associated CRESS DNA virus 2, 84-AMS-01	MT293425	Vilyaviridae	Giardia intestinalis assemblage A WGS project CAHQ00000000	CAHQ01000943.1	30	81.49	1.00E-171
Giardia-associated CRESS DNA virus 2, 84-AMS-02	MT293426	Vilyaviridae	Giardia intestinalis assemblage A WGS project CAHQ00000000	CAHQ01001040.1	44	72.16	6.00E-125
Giardia-associated CRESS DNA virus 3 CDS, 84-AMS-01	MT293427	Vilyaviridae	Giardia intestinalis ATCC 50581 strain GS/M clone H7 contig2341, whole genome shotgun sequence	ACGJ01002178.1	75	73.07	0
Giardia-associated CRESS DNA virus 3, 84-AMS-02	MT293428	Vilyaviridae	Giardia intestinalis ATCC 50581 strain GS/M clone H7 contig3001, whole genome shotgun sequence	ACGJ01002837.1	60	76.33	0
Giardia-associated CRESS DNA virus 4, 84-AMS-01	MT293429	Vilyaviridae	Giardia intestinalis isolate AS98 genome assembly, contig: AS98_contig22, whole genome shotgun sequence	CVLA01000027.1	66	67.19	6.00E-80

Supplementary Table 6. CRESS integrations in *Entamoeba* are shared among multiple independent genome assemblies. Sequences from endogenous viral elements (columns) were queried against available assemblies of *E. histolytica* (rows) using BLASTn, revealing a wide distribution among independent parasite isolates (implying these are not artefacts of genome assemblies). ND = not done (since the assembly was also the source of the integration), NS = not significant (1e-10 cutoff).

		**Endogenous viral element query sequences**								
		E. histolytica isolate	HM-1:IMSS	HM-1:IMSS	HM-1:IMSS	KU27	HM-1:IMSS	HM-1:IMSS	HM1:IMSS	KU27
		Molecule type	DNA	DNA	DNA	DNA	DNA	RNA	DNA	DNA
		Isolation place and year	Mexico, 1967	Mexico, 1967	Mexico, 1967	Japan, 2001	Mexico, 1967	Mexico, 1967	Mexico, 1967	Japan, 2001
**Subject assemblies**	**Information**	Assembly accession	GCA_000208925.2	GCA_000208925.2	GCA_000208925.2	GCA_000338855.1	GCA_000208925.2	NA	GCA_001662325.1	GCA_000338855.1
(accession,	(reference,	Contig accession	NW_001915013.1	NW_001915013.1	NW_001915253.1	KB445521.1	NW_001915135.1	CX089530.1	BDEQ01000001.1	KB445272.1
isolate,	assembly total length,	EVE protein accession	XP_652805.2	XP_652803.1	XP_648754.1	EMD42418.1	XP_001914113.1	NA	GAT95322.1	EMD43492.1
place and year of isolation)	contig count & contig N50)	EVE nucleotide accession	XM_647713.2	XM_647711.1	XM_643662.1	NA (nt sequence is partial)	XM_001914078.1	CX089530.1	NA	NA
GCA_000208925.2	Lorenzi et al 2010	Best hit contig	ND	ND	ND	NW_001915456.1	ND	NW_001915456.1	NW_001914934.1	NW_001914934.1
E. histolytica HM-1:IMSS	20.8 Mb	Query coverage	ND	ND	ND	100	ND	100	100	100
Mexico, 1967	2,172 (29 Kb)	Identity	ND	ND	ND	99.44	ND	99.64	100	99.89
GCA_001662325.1		Best hit contig	BDEQ01000001.1	BDEQ01000001.1	BDEQ01000001.1	BDEQ01000001.1	BDEQ01000001.1	BDEQ01000001.1	ND	BDEQ01000001.1
E. histolytica HM1:IMSS clone 6	19.9 Mb	Query coverage	100	100	100	100	100	100	ND	100
Mexico, 1967	NA (concatenated)	Identity	88.67	100	99.83	99.44	100	99.64	ND	99.89
GCA_000365475.1		Best hit contig	NS	NS	NS	NS	NS	NS	KB823243.1	KB823243.1
E. histolytica HM-1:IMSS-A	12.3 Mb	Query coverage	NS	NS	NS	NS	NS	NS	100	100
Lab strain, Germany	1,691 (9 Kb)	Identity	NS	NS	NS	NS	NS	NS	100	99.89
GCA_000338855.1		Best hit contig	KB445248.1	KB443944.1	KB445521.1	ND	KB445521.1	KB445521.1	KB445272.1	ND
E. histolytica KU27	15.3 Mb	Query coverage	100	100	100	ND	100	100	100	ND
Japan, 2001	3,228 (9 Kb)	Identity	88.67	100	99.33	ND	99.44	99.52	99.89	ND
GCA_000344925.1		Best hit contig	NS	NS	NS	NS	NS	NS	NS	NS
E. histolytica HM-1:IMSS-B	12.8 Mb	Query coverage	NS	NS	NS	NS	NS	NS	NS	NS
Lab strain, Germany	2,283 (8.6 Kb)	Identity	NS	NS	NS	NS	NS	NS	NS	NS
GCA_000346345.1		Best hit contig	KB638588.1	NS	KB637742.1	KB637742.1	KB637742.1	KB637742.1	KB638686.1	KB638686.1
E. histolytica HM-3:IMSS	13.8 Mb	Query coverage	100	NS	100	100	100	98	100	100
Mexico, 1972	3,428 (7 Kb)	Identity	88.55	NS	99.5	99.44	100	99.51	99.89	100

Supplementary Table 7. CRESS integrations in *Giardia* are shared among multiple independent genome assemblies. Sequences from endogenous viral elements (columns) were queried against available assemblies of *G. duodenalis* (rows) using BLASTn, revealing a wide but lineage-specific distribution among independent parasite isolates (implying these are not artefacts of genome assemblies). ND = not done (since the assembly was also the source of the integration), NS = not significant (1e-10 cutoff).

		**Endogenous viral element query sequences**										
		G. duodenalis isolate	DH	DH	BRIS/92/HEPU/1541	BRIS/92/HEPU/1541	GS	GS	GS	GS	BAH15c1	GS
		Molecule type	DNA	DNA	DNA	DNA	DNA	DNA	DNA	DNA	DNA	DNA
		Isolation place and year	West Virginia, USA, 1985	West Virginia, USA, 1985	Australia, 1992	Australia, 1992	Alaska, USA, 1985	Alaska, USA, 1985	Alaska, USA, 1985	Alaska, USA, 1985	Australia	Alaska, USA, 1985
**Subject assemblies**	**Information**	Assembly accession	GCA_000498715.1	GCA_000498715.1	NA	NA	GCA_000182405.1	GCA_000498735.1	GCA_000498735.1	GCA_000182405.1	GCA_001543975.1	GCA_000498735.1
(accession,	(reference,	Contig accession	AHGT01000120.1	AHGT01000120.1	AF059664.1	AF059664.1	ACGJ01002837.1	AHHH01000265.1	AHHH01000265.1	ACGJ01002178.1	JXTI01000402.1	AHHH01000424.1
isolate & lineage,	assembly total length,	EVE protein accession	ESU34896.1	ESU34895.1	AAF28772.1	AAF28770.1	EES99432.1	ESU40270.1	ESU40268.1	EET01110.1	KWX11074.1	ESU39907.1
place and year of isolation)	contig count & contig N50)	EVE nucleotide accession	NA	NA	NA	NA	NA	NA	NA	NA	NA	NA
GCA_000002435.2	Xu et al. 2020.	Best hit contig	NS	NS	NS	NS	NS	NS	NS	NS	NS	NS
G. duodenalis WB (lineage A1)	12.1 Mb	Query coverage	NS	NS	NS	NS	NS	NS	NS	NS	NS	NS
Maryland, USA, 1979	38 (2,761 Kb)	Identity	NS	NS	NS	NS	NS	NS	NS	NS	NS	NS
GCA_009192825.1	Weisz et al. 2019.	Best hit contig	NS	NS	NS	NS	NS	NS	NS	NS	NS	NS
G. duodenalis ZX15 (lineage A1)	10.3 Mb	Query coverage	NS	NS	NS	NS	NS	NS	NS	NS	NS	NS
Czech Republic, 2015	611 (57 Kb)	Identity	NS	NS	NS	NS	NS	NS	NS	NS	NS	NS
GCA_011634545.1	Pollo et al. 2020	Best hit contig	NS	NS	NS	NS	NS	NS	NS	NS	NS	NS
G. duodenalis WB (lineage A1)	11.7 Mb	Query coverage	NS	NS	NS	NS	NS	NS	NS	NS	NS	NS
Maryland, USA, 1979	37 (616 Kb)	Identity	NS	NS	NS	NS	NS	NS	NS	NS	NS	NS
GCA_000498715.1	Adam et al. 2013.	Best hit contig	ND	ND	AHGT01000120.1	AHGT01000120.1	AHGT01000120.1	AHGT01000120.1	AHGT01000120.1	AHGT01000120.1	NS	NS
G. duodenalis DH (lineage A2)	10.7 Mb	Query coverage	ND	ND	100	100	43	33	40	41	NS	NS
West Virginia, USA, 1985	239 (117 Kb)	Identity	ND	ND	99.67	99.83	75.73	73.26	74.6	73.81	NS	NS
GCA_001493575.1	Ankarklev et al. 2015	Best hit contig	CAHQ01000875.1	CAHQ01000943.1	CAHQ01000875.1	CAHQ01000943.1	CAHQ01001040.1	CAHQ01001083.1	CAHQ01001083.1	CAHQ01001083.1	NS	NS
G. duodenalis AS175 (lineage A2)	10.5 Mb	Query coverage	100	96	100	96	77	39	40	42	NS	NS
Sweden	1139 (57 Kb)	Identity	99.89	99.46	99.78	99.28	71.29	72.88	75.93	74.94	NS	NS
CVLA00000000.1	Ankarklev et al. 2015	Best hit contig	CVLA01000027.1	CVLA01000027.1	CVLA01000027.1	CVLA01000027.1	NS	NS	NS	NS	NS	NS
G. duodenalis AS98 (lineage A2)	10.3 Mb	Query coverage	100	100	100	100	NS	NS	NS	NS	NS	NS
Sweden	914 (43Kb)	Identity	99.89	99.83	99.78	99.65	NS	NS	NS	NS	NS	NS
GCA_000182405.1	Franzén et al. 2009	Best hit contig	ACGJ01000559.1	ACGJ01002837.1	ACGJ01000559.1	ACGJ01002837.1	ND	ACGJ01002500.1	ACGJ01002178.1	ND	ACGJ01002413.1	ACGJ01002413.1
G. duodenalis GS (lineage B)	11 Mb	Query coverage	34	65	33	65	ND	94	92	ND	100	100
Alaska, USA, 1985	2931 (34 Kb)	Identity	68.73	75.73	68.73	75.47	ND	99.28	99.07	ND	94.87	100
GCA_000498735.1	Adam et al. 2013	Best hit contig	NS	AHHH01000265.1	NS	AHHH01000265.1	AHHH01000413.1	ND	ND	AHHH01000265.1	AHHH01000424.1	ND
G. duodenalis GS (lineage B)	12 Mb	Query coverage	NS	65	NS	65	38	ND	ND	100	100	ND
Alaska, USA, 1985	543 (58 Kb)	Identity	NS	74.6	NS	74.34	92.74	ND	ND	99.07	94.87	ND
GCA_011634595.1	Pollo et al. 2020	Best hit contig	VSRU01000007.1	VSRU01000007.1	VSRU01000007.1	VSRU01000007.1	VSRU01000007.1	VSRU01000012.1	VSRU01000012.1	VSRU01000012.1	VSRU01000009.1	VSRU01000009.1
G. duodenalis GS (lineage B)	13.2 Mb	Query coverage	34	65	33	65	100	100	100	100	100	100
Alaska, USA, 1985	19 (1,645 Kb)	Identity	68.95	76.06	68.95	75.8	99.78	99.91	100	100	94.87	100
GCA_001543975.1	Wielinga et al. 2015	Best hit contig	NS	NS	NS	NS	NS	NS	NS	NS	ND	JXTI01000402.1
G. duodenalis BAH15c1 (lineage B)	10.4 Mb	Query coverage	NS	NS	NS	NS	NS	NS	NS	NS	ND	100
Australia	708 (33 Kb)	Identity	NS	NS	NS	NS	NS	NS	NS	NS	ND	94.87
GCA_000182665.1	Jerlström-Hultqvist et al. 2010	Best hit contig	NS	NS	NS	NS	NS	NS	NS	NS	NS	NS
G. duodenalis P15 (lineage E)	11.5 Mb	Query coverage	NS	NS	NS	NS	NS	NS	NS	NS	NS	NS
Czech Republic, 1991	820 (71 Kb)	Identity	NS	NS	NS	NS	NS	NS	NS	NS	NS	NS

Supplementary Table 8. Delimitation of CRESS virus genera. Genera infecting the same host genus were assigned a Greek number and named with reference to the host. Pairwise comparisons of Rep proteins were done using SIAS (available at: http://imed.med.ucm.es/Tools/sias.html), using mean length of the sequences as the denominator. A cutoff of 50% protein identity was used for genus delimitation, following a recent literature example[8].

Family	Virus	Genus	Sequence number	*S1*	*S2*	*S3*	*S4*	*S5*	*S6*	*S7*	*S8*	*S9*	*S10*	*S11*	*S12*	*S13*	*S14*	*S15*	*S16*	*S17*	*S18*	*S19*	*S20*
Naryaviridae	Entamoeba-associated CRESS DNA virus 1, 84-AMS-01 rep	Protoentvirus	*S1*	**100**	**93**	**93**	**77**	42	42	21	20	19	19	19	21	20	20	21	25	25	23	21	20
Naryaviridae	Entamoeba-associated CRESS DNA virus 1, 84-AMS-02 rep	Protoentvirus	*S2*	**93**	**100**	**100**	**77**	41	41	22	21	19	19	19	21	20	20	20	25	25	22	21	19
Naryaviridae	Entamoeba-associated CRESS DNA virus 1, 94-AMS-01 rep	Protoentvirus	*S3*	**93**	**100**	**100**	**77**	41	41	22	21	19	19	19	21	20	20	20	25	25	22	21	19
Naryaviridae	Entamoeba-associated CRESS DNA virus 1, 84-AMS-03 rep	Protoentvirus	*S4*	**77**	**77**	**77**	**100**	42	43	19	19	20	20	20	19	21	21	21	24	24	21	20	19
Naryaviridae	Entamoeba-associated CRESS DNA virus 2, 84-AMS-01 rep	Deuteroentvirus	*S5*	42	41	41	42	**100**	**100**	21	21	22	22	22	19	20	20	20	21	21	22	19	18
Naryaviridae	Entamoeba-associated CRESS DNA virus 2, 94-AMS-01 rep	Deuteroentvirus	*S6*	42	41	41	43	**100**	**100**	21	21	22	22	22	19	20	20	20	21	21	22	19	18
Nenyaviridae	Entamoeba-associated CRESS DNA virus 3, 84-AMS-01 rep	Tritoentvirus	*S7*	21	22	22	19	21	21	**100**	**94**	48	48	48	21	22	22	22	23	23	22	24	25
Nenyaviridae	Entamoeba-associated CRESS DNA virus 3, 84-AMS-02 rep	Tritoentvirus	*S8*	20	21	21	19	21	21	**94**	**100**	46	47	47	21	22	22	22	23	23	22	24	25
Nenyaviridae	Entamoeba-associated CRESS DNA virus 4, 84-AMS-01 rep	Tetartoentvirus	*S9*	19	19	19	20	22	22	48	46	**100**	**100**	**100**	22	22	22	22	23	23	19	22	22
Nenyaviridae	Entamoeba-associated CRESS DNA virus 4, 84-AMS-02 rep	Tetartoentvirus	*S10*	19	19	19	20	22	22	48	47	**100**	**100**	**100**	22	22	22	22	23	23	19	22	22
Nenyaviridae	Entamoeba-associated CRESS DNA virus 4, 84-AMS-03 rep	Tetartoentvirus	*S11*	19	19	19	20	22	22	48	47	**100**	**100**	**100**	22	22	22	22	23	23	19	22	22
Vilyaviridae	Giardia-associated CRESS DNA virus 1 CDS, 84-AMS-04 rep	Protogiavirus	*S12*	21	21	21	19	19	19	21	21	22	22	22	**100**	**68**	**68**	**68**	51	51	49	41	38
Vilyaviridae	Giardia-associated CRESS DNA virus 1, 84-AMS-01 rep	Protogiavirus	*S13*	20	20	20	21	20	20	22	22	22	22	22	**68**	**100**	**100**	**99**	49	49	49	48	44
Vilyaviridae	Giardia-associated CRESS DNA virus 1, 84-AMS-02 rep	Protogiavirus	*S14*	20	20	20	21	20	20	22	22	22	22	22	**68**	**100**	**100**	**99**	49	49	49	48	44
Vilyaviridae	Giardia-associated CRESS DNA virus 1, 84-AMS-03 rep	Protogiavirus	*S15*	21	20	20	21	20	20	22	22	22	22	22	**68**	**99**	**99**	**100**	50	50	49	49	45
Vilyaviridae	Giardia-associated CRESS DNA virus 3 CDS, 84-AMS-01 rep	Deuteroentvirus	*S16*	25	25	25	24	21	21	23	23	23	23	23	51	49	49	50	**100**	**100**	**62**	43	43
Vilyaviridae	Giardia-associated CRESS DNA virus 3, 84-AMS-02 rep	Deuteroentvirus	*S17*	25	25	25	24	21	21	23	23	23	23	23	51	49	49	50	**100**	**100**	**61**	43	43
Vilyaviridae	Giardia-associated CRESS DNA virus 4, 84-AMS-01 rep	Deuteroentvirus	*S18*	23	22	22	21	22	22	22	22	19	19	19	49	49	49	49	**62**	**61**	**100**	40	40
Vilyaviridae	Giardia-associated CRESS DNA virus 2, 84-AMS-01 rep	Tritogiavirus	*S19*	21	21	21	20	19	19	24	24	22	22	22	41	48	48	49	43	43	40	**100**	**84**
Vilyaviridae	Giardia-associated CRESS DNA virus 2, 84-AMS-02 rep	Tritogiavirus	*S20*	20	19	19	19	18	18	25	25	22	22	22	38	44	44	45	43	43	40	**84**	**100**

Supplementary Table 9. Host and virus GC-content. Host-virus relationships were found using the Virus-Host DB, and GC-content was retrieved from genome assemblies available in the NCBI genome assemblies database.

CRESS family	Virus species	Accession(s)	Virus GC percentage	Selected reference host	Host accession	Host GC percentage
Naryaviridae	Entamoeba-associated CRESS DNA virus 1, 84-AMS-01	MT293410	36.84	Entamoeba histolytica	AAFB00000000.2	25.20
Naryaviridae	Entamoeba-associated CRESS DNA virus 1, 84-AMS-02	MT293411	37.03	Entamoeba histolytica	AAFB00000000.2	25.20
Naryaviridae	Entamoeba-associated CRESS DNA virus 1, 94-AMS-01	MT293412	37.52	Entamoeba histolytica	AAFB00000000.2	25.20
Naryaviridae	Entamoeba-associated CRESS DNA virus 1, 84-AMS-03	MT293413	35.05	Entamoeba histolytica	AAFB00000000.2	25.20
Naryaviridae	Entamoeba-associated CRESS DNA virus 2, 84-AMS-01	MT293414	36.74	Entamoeba histolytica	AAFB00000000.2	25.20
Naryaviridae	Entamoeba-associated CRESS DNA virus 2, 94-AMS-01	MT293415	37.36	Entamoeba histolytica	AAFB00000000.2	25.20
Nenyaviridae	Entamoeba-associated CRESS DNA virus 3, 84-AMS-01	MT293416	42.75	Entamoeba histolytica	AAFB00000000.2	25.20
Nenyaviridae	Entamoeba-associated CRESS DNA virus 3, 84-AMS-02	MT293417	42.74	Entamoeba histolytica	AAFB00000000.2	25.20
Nenyaviridae	Entamoeba-associated CRESS DNA virus 4, 84-AMS-01	MT293418	41.84	Entamoeba histolytica	AAFB00000000.2	25.20
Nenyaviridae	Entamoeba-associated CRESS DNA virus 4, 84-AMS-02	MT293419	39.86	Entamoeba histolytica	AAFB00000000.2	25.20
Nenyaviridae	Entamoeba-associated CRESS DNA virus 4, 84-AMS-03	MT293420	42.05	Entamoeba histolytica	AAFB00000000.2	25.20
Nenyaviridae	Unidentified circular ssDNA virus clone V16C Rep and hypothetical protein genes	MG571899	44.14	Entamoeba histolytica	AAFB00000000.2	25.20
Nenyaviridae	Circoviridae sp. isolate ctga69, complete genome	MH617639	40.36	Entamoeba histolytica	AAFB00000000.2	25.20
Nenyaviridae	Unidentified circular ssDNA virus isolate cg6319, complete genome	KU043415	34.17	Entamoeba histolytica	AAFB00000000.2	25.20
Vilyaviridae	Giardia-associated CRESS DNA virus 1, 84-AMS-01	MT293421	60.54	Giardia duodenalis	NZ_AACB00000000.2	48.20
Vilyaviridae	Giardia-associated CRESS DNA virus 1, 84-AMS-02	MT293422	60.57	Giardia duodenalis	NZ_AACB00000000.2	48.20
Vilyaviridae	Giardia-associated CRESS DNA virus 1, 84-AMS-03	MT293423	61.10	Giardia duodenalis	NZ_AACB00000000.2	48.20
Vilyaviridae	Giardia-associated CRESS DNA virus 2, 84-AMS-01	MT293425	59.89	Giardia duodenalis	NZ_AACB00000000.2	48.20
Vilyaviridae	Giardia-associated CRESS DNA virus 2, 84-AMS-02	MT293426	53.25	Giardia duodenalis	NZ_AACB00000000.2	48.20
Vilyaviridae	Giardia-associated CRESS DNA virus 3, 84-AMS-02	MT293428	55.82	Giardia duodenalis	NZ_AACB00000000.2	48.20
Vilyaviridae	Giardia-associated CRESS DNA virus 4, 84-AMS-01	MT293429	60.47	Giardia duodenalis	NZ_AACB00000000.2	48.20
Vilyaviridae	Uncultured virus clone CG97, complete genome	KY487991	55.33	Giardia duodenalis	NZ_AACB00000000.2	48.20
Vilyaviridae	Feline stool-associated circular virus KU7 DNA, complete genome	LC406405	57.67	Giardia duodenalis	NZ_AACB00000000.2	48.20
Alphasatellitidae	Chilli leaf curl India alphasatellite	NC_027431	40.87	Capsicum annuum	GCA_000710875.1	35.41
Alphasatellitidae	Chilli leaf curl Multan alphasatellite	NC_013103	40.69	Capsicum annuum	GCA_000710875.1	35.41
Alphasatellitidae	Papaya leaf curl alphasatellite clone PaLCuA[PK/Mul/07]	NC_023292	40.47	Carica papaya	GCA_000150535.1	39.01

Alphasatellitidae	Coconut foliar decay alphasatellite 3	NC_040624	39.62	Cocos nucifera	GCA_006176705.1	37.60
Alphasatellitidae	Coconut foliar decay alphasatellite 6	NC_040697	41.61	Cocos nucifera	GCA_006176705.1	37.60
Alphasatellitidae	Cucurbit yellow mosaic alphasatellite isolate 51SA	NC_029907	40.15	Cucurbita pepo	GCA_002806865.2	37.26
Alphasatellitidae	Cotton leaf curl Burewala alphasatellite	NC_013803	42.31	Gossypium hirsutum	GCA_000987745.1	34.90
Alphasatellitidae	Cotton leaf curl virus associated alphasatellite	KM070823	40.70	Gossypium hirsutum	GCA_000987745.1	34.90
Alphasatellitidae	Cotton leaf curl virus associated DNA 1 isolate Lucknow	NC_015327	43.05	Gossypium hirsutum	GCA_000987745.1	34.90
Alphasatellitidae	Hibiscus leaf curl alphasatellite	NC_033273	40.07	Hibiscus syriacus	GCA_006381635.1	34.30
Alphasatellitidae	Cassava mosaic Madagascar alphasatellite	NC_018628	44.93	Manihot esculenta	GCA_001659605.1	38.01
Alphasatellitidae	Banana bunchy top alphasatellite 1	NC_038892	43.34	Musa acuminata	GCA_000313855.2	40.73
Alphasatellitidae	Banana bunchy top alphasatellite 2	NC_038953	43.64	Musa acuminata	GCA_000313855.2	40.73
Alphasatellitidae	Subterranean clover stunt C2	NC_003814	42.07	Trifolium subterraneum	GCA_001742945.1	35.20
Alphasatellitidae	Subterranean clover stunt C6 alphasatellite	NC_003818	41.79	Trifolium subterraneum	GCA_001742945.1	35.20
Alphasatellitidae	Faba bean necrotic yellows C1 alphasatellite	NC_024886	39.32	Vicia faba	GCA_001375635.1	38.90
Alphasatellitidae	Faba bean necrotic yellows C11 alphasatellite	NC_003558	40.14	Vicia faba	GCA_001375635.1	38.90
Circoviridae	Anguilla anguilla circovirus	NC_023421	48.69	Anguilla anguilla	GCA_000695075.1	42.90
Circoviridae	Pigeon circovirus strain SF079/ShangHai/2014	KX108827	55.36	Columba livia	GCA_000337935.1	41.60
Circoviridae	Beak and feather disease virus natural-host Cacatua galerita	AF311301	52.76	Melopsittacus undulatus	NC_009134.1	41.40
Circoviridae	Beak and feather disease virus natural-host Cacatua leabeateri	AF311300	53.68	Melopsittacus undulatus	NC_009134.1	41.40
Circoviridae	Beak and feather disease virus natural-host Trichoglossus haematodus	AF311299	53.71	Melopsittacus undulatus	NC_009134.1	41.40
Circoviridae	Canary circovirus	NC_003410	51.28	Serinus canaria	GCA_000534875.1	42.60
Circoviridae	Porcine circovirus 2 isolate AUT1	AY424401	48.53	Sus scrofa	GCA_000003025.6	41.97
Geminiviridae	Beet curly top Iran virus-[Siv]	JX082259	40.60	Beta vulgaris	GCA_000511025.2	37.30
Geminiviridae	Chilli leaf curl virus-Noida	HM140371	44.03	Capsicum annuum	GCA_000710875.1	35.41
Geminiviridae	Pepper leaf curl Yunnan virus-[YN323]	NC_010618	41.72	Capsicum annuum	GCA_000710875.1	35.41
Geminiviridae	Papaya leaf crumple virus complete genome	HE580236	44.36	Carica papaya	GCA_000150535.1	39.01
Geminiviridae	Papaya leaf curl China virus GX4 complete genome	FN297834	41.97	Carica papaya	GCA_000150535.1	39.01
Geminiviridae	Chickpea chlorosis Australia virus isolate CpCV-D_AU_3494I_2002	NC_022131	39.89	Cicer arietinum	GCA_000331145.1	32.67
Geminiviridae	Chickpea chlorosis virus isolate F 3487J	KC172700	43.05	Cicer arietinum	GCA_000331145.1	32.67
Geminiviridae	Chickpea yellow dwarf virus isolate CpYDV-	NC_025475	41.03	Cicer arietinum	GCA_000331145.1	32.67

	PK_PK103_2012					
Geminiviridae	Squash leaf curl virus	NC_001936-37	44.06	Cucurbita pepo	GCA_002806865.2	37.26
Geminiviridae	Squash yellow mild mottle virus	NC_003860, NC_003865	43.02	Cucurbita pepo	GCA_002806865.2	37.26
Geminiviridae	Cotton leaf curl Alabad virus	NC_004582	42.75	Gossypium hirsutum	GCA_000987745.1	34.90
Geminiviridae	Cotton leaf curl Multan virus-[Faisalabad2]	AJ496287	43.71	Gossypium hirsutum	GCA_000987745.1	34.90
Geminiviridae	Abutilon mosaic virus	NC_001928-29	46.71	Hibiscus syriacus	GCA_006381635.1	34.30
Geminiviridae	Barley dwarf virus complete genome, isolate DE45BDV	HF968650	49.23	Hordeum vulgare	GCA_000326125.1	44.90
Geminiviridae	Apple geminivirus PL-2015	NC_026760	45.57	Malus domestica	GCA_002114115.1	39.36
Geminiviridae	African cassava mosaic virus	GCA_000857205	42.13	Manihot esculenta	GCA_001659605.1	38.01
Geminiviridae	Tomato pseudo-curly top virus	NC_003825	41.60	Solanum lycopersicum	GCA_000188115.3	35.70
Geminiviridae	Tomato yellow vein streak virus	NC_010949-50	39.47	Solanum lycopersicum	GCA_000188115.3	35.70
Geminiviridae	Potato yellow mosaic virus	NC_001934-35	43.31	Solanum tuberosum	GCA_000226075.1	35.60
Geminiviridae	Wheat dwarf virus - [Sweden]	X02869	47.72	Triticum aestivum	GCA_900067645.1	43.50
Geminiviridae	Cowpea golden mosaic virus-[Nigeria] DNA-A	NC_038445	45.57	Vigna unguiculata	GCA_004118075.1	33.06
Geminiviridae	Wild vitis virus 1 isolate WVV1-NY1468	NC_035480	42.25	Vitis vinifera	GCA_000003745.2	35.03
Geminiviridae	Maize streak virus - E[Pat] isolate MSV-Pat	AF329888	49.61	Zea mays	GCA_000005005.6	46.91
Genomoviridae	Sclerotinia sclerotiorum hypovirulence associated DNA virus 1	GQ365709	47.22	Sclerotinia sclerotiorum	AAGT00000000.1	41.70
Genomoviridae	Sclerotinia sclerotiorum hypovirulence associated DNA virus 1	KM598384	46.81	Sclerotinia sclerotiorum	AAGT00000000.1	41.70
Nanoviridae	Abaca bunchy top virus	NC_010314-19	41.05	Musa acuminata	GCA_000313855.2	40.73
Nanoviridae	Banana bunchy top virus	NC_003473-77, NC_003479	40.38	Musa acuminata	GCA_000313855.2	40.73
Nanoviridae	Pea necrotic yellow dwarf virus	GCA_000914235	40.10	Pisum sativum	PUCA000000000.1	37.70
Nanoviridae	Pea yellow stunt virus	GCA_000915955	41.25	Pisum sativum	PUCA000000000.1	37.70
Nanoviridae	Subterranean clover stunt virus	NC_003812-13, NC_003815-17, NC_003819	38.81	Trifolium subterraneum	GCA_001742945.1	35.20
Nanoviridae	Faba bean yellow leaf virus	NC_038908	37.32	Vicia faba	GCA_001375635.1	38.90
Smacoviridae	Human smacovirus 1 isolate France/12/2008/3454	NC_026252	43.94	Candidatus Methanomassiliicoccus intestinalis	NC_021353.1	41.30

Supplementary Table 10. Primer and probe sequences used in this study.

Name	Sequence	Description
F11_A1_F1	GCAAGACGCTTCCAACTTACGC	MT293412 inverse PCR amplicon 1
F11_A1_R1	GCAGGATTCAAAGCAAATGCAGC	MT293412 inverse PCR amplicon 1
F11_A2_F1	CGAGAATTTGGCTTCAGAGTGG	MT293412 inverse PCR amplicon 2
F11_A2_R1	CGTCTTCCTCCTTATGCAATC	MT293412 inverse PCR amplicon 2
F11_A3_F1	GCTTCAGACAGGGGATTCTG	MT293412 inverse PCR amplicon 3
F11_A3_R1	GGAAGGCTTCATGTCAGAG	MT293412 inverse PCR amplicon 3
F95_A1_F1	CGTTACAATACTTGACTCGATC	MT293415 inverse PCR amplicon 1 round 1
F95_A1_R1	GACTGGAGTTTCTAGGGATG	MT293415 inverse PCR amplicon 1 round 1
F95_A1_F2	GCTCAAGTTCACGTAATTTATC	MT293415 inverse PCR amplicon 1 round 2
F95_A1_R2	GACTCAGTAAGGCTGCTTGG	MT293415 inverse PCR amplicon 1 round 2
F95_A2_F1	GCTGGTTCTGCATTATGTTTG	MT293415 inverse PCR amplicon 2 round 1
F95_A2_R1	CATAATATCTGTTCGTATAACG	MT293415 inverse PCR amplicon 2 round 1
F95_A2_F2	CTTCACAAAGTGTTTCTACTG	MT293415 inverse PCR amplicon 2 round 2
F95_A2_R2	CTTCTGTAATAACCTCTAGCC	MT293415 inverse PCR amplicon 2 round 2
F11_A1_F2	CTTAACCAACCAGACGAATATG	Filtrate screening PCR
F11_A3_R1	GGAAGGCTTCATGTCAGAG	Filtrate screening PCR
S7072_A1_F1	CCAGTTGACTCTTAACCAGC	MT293413 amplification PCR amplicon 1
S7072_A1_R1	CATCCTTCTTAGCCACTGCTTTC	MT293413 amplification PCR amplicon 1
S7072_A2_F1	GAAAATCGAAGAAAGTCTTTG	MT293413 amplification PCR amplicon 2
S7072_A2_R1	GTAATTATTTGAGGGAGACTATC	MT293413 amplification PCR amplicon 2
S7072_A3_F1	TTCATCTTAGAAGTTTAGAAG	MT293413 amplification PCR amplicon 3
S7072_A3_R1	GTTTTAACAGAAGGTATCCATTT	MT293413 amplification PCR amplicon 3
S7072_A4_F1	GGCTGGCTATACTACTCTCA	MT293413 amplification PCR amplicon 4
S7072_A4_R1	CATATAAGGCTTAACATAGAAC	MT293413 amplification PCR amplicon 4
S7072_A5_F1	ATCCTGAAGATGATTCTAAAC	MT293413 amplification PCR amplicon 5
S7072_A5_R1	CCATGTAGCAGTTTGAGAAG	MT293413 amplification PCR amplicon 5
S7072_A6_F1	GAAGAGGATGAGATATGTTAAG	MT293413 amplification PCR amplicon 6
S7072_A6_R1	TGTTCAGGCTGGTTAAGAGTC	MT293413 amplification PCR amplicon 6
Ent_18S_F1	GTTGATCCTGCCAGTATT	Entamoeba generic 18S PCR
Ent_18S_R1	GAGCTGGAATTACCGC	Entamoeba generic 18S PCR
Ent_18S_F2	CCTGCCAGTATTATATGC	Entamoeba generic 18S PCR
Ent_18S_R2	GCACCAGACTTGCCCTCC	Entamoeba generic 18S PCR
E_histolytica_F1	ATTgTCgTggCATCCTAACTCA	Entamoeba histolytica diagnostic qPCR
E_histolytica_R1	gCggACggCTCATTATAACA	Entamoeba histolytica diagnostic qPCR
E_histolytica_probe	UCAUUGAAUGAAUUGGCCAUUU	Entamoeba histolytica diagnostic qPCR
G_duodenalis_F1	GACGGCTCAGGACAACGGTT	Giardia duodenalis diagnostic qPCR
G_duodenalis_R1	TTGCCAGCGGTGTCCG	Giardia duodenalis diagnostic qPCR
G_duodenalis_probe	CCCGCGGCGGTCCCTGCTAG	Giardia duodenalis diagnostic qPCR
D_fragilis_F1	CAACGGATGTCTTGGCTCTTTA	Dientamoeba fragilis diagnostic qPCR
D_fragilis_R1	TGCATTCAAAGATCGAACTTATCAC	Dientamoeba fragilis diagnostic qPCR
D_fragilis_probe	CAATTCTAGCCGCTTAT	Dientamoeba fragilis diagnostic qPCR
Cry_spp_F1	CGCTTCTCTAGCCTTTCATGA	Cryptosporidium diagnostic qPCR
Cry_spp_R1	CTTCACGTGTGTTTGCCAAT	Cryptosporidium diagnostic qPCR
Cry_spp_probe	CCAATCACAGAATCATCAGAATCGACTGGTATC	Cryptosporidium diagnostic qPCR
Blast_spp_F1	CGTTGTTGCAGTTAAAAAGCTCGT	Blastocystis diagnostic qPCR
Blast_spp_R1	GATTAATGAAAACATCCTTGGTAAATGC	Blastocystis diagnostic qPCR
Blast_spp_probe	CAGTTGGGGGTATTCATATTC	Blastocystis diagnostic qPCR

Supplementary References

1. Pollo, S. M. J. *et al.* Benchmarking hybrid assemblies of Giardia and prediction of widespread intra-isolate structural variation. *Parasites and Vectors* **13**, 108 (2020).

2. Krzywinski, M. *et al.* Circos: an information aesthetic for comparative genomics. *Genome Res.* **19**, 1639–45 (2009).

3. Krupovic, M. & Forterre, P. Single-stranded DNA viruses employ a variety of mechanisms for integration into host genomes. *Ann. N. Y. Acad. Sci.* **1341**, 41–53 (2015).

4. Zhang, H., Ehrenkaufer, G. M., Hall, N. & Singh, U. Small RNA pyrosequencing in the protozoan parasite *Entamoeba histolytica* reveals strain-specific small RNAs that target virulence genes. *BMC Genomics* **14** (2013).

5. Heyraud, F., Matzeit, V., Schaefer, S., Schell, J. & Gronenborn, B. The conserved nonanucleotide motif of the geminivirus stem-loop sequence promotes replicational release of virus molecules from redundant copies. *Biochimie* **75**, 605–615 (1993).

6. Zuker, M. Mfold web server for nucleic acid folding and hybridization prediction. *Nucleic Acids Res.* **31**, 3406–3415 (2003).

7. Potter, S. C. *et al.* HMMER web server: 2018 update. *Nucleic Acids Res.* **46**, W200–W204 (2018).

8. Abbas, A. A. *et al. Redondoviridae*, a family of small, circular DNA viruses of the human oro-respiratory tract that are associated with periodontitis and critical illness. *Cell Host Microbe* **25**, 719–729 (2019).
