# Supplementary material for: Entamoeba and Giardia parasites implicated as hosts of CRESS viruses
Source: Nat Commun. 2020 Sep 15;11:4620. doi: 10.1038/s41467-020-18474-w (PMC7493932; doi:10.1038/s41467-020-18474-w)
Supplement: Supplementary file 2 — Reporting Summary [file 41467_2020_18474_MOESM2_ESM.pdf]

nature research

Corresponding author(s): Cormac M. Kinsella
Lia van der Hoek

Last updated by author(s): Aug 21, 2020

Reporting Summary

Nature Research wishes to improve the reproducibility of the work that we publish. This form provides structure for consistency and transparency in reporting. For further information on Nature Research policies, see Authors & Referees and the Editorial Policy Checklist .

Statistics

For all statistical analyses, confirm that the following items are present in the figure legend, table legend, main text, or Methods section.

n/a	Confirmed	
☐	☒	The exact sample size (n) for each experimental group/condition, given as a discrete number and unit of measurement
☒	☐	A statement on whether measurements were taken from distinct samples or whether the same sample was measured repeatedly
☐	☒	The statistical test(s) used AND whether they are one- or two-sided<br>*Only common tests should be described solely by name; describe more complex techniques in the Methods section.*
☒	☐	A description of all covariates tested
☒	☐	A description of any assumptions or corrections, such as tests of normality and adjustment for multiple comparisons
☒	☐	A full description of the statistical parameters including central tendency (e.g. means) or other basic estimates (e.g. regression coefficient) AND variation (e.g. standard deviation) or associated estimates of uncertainty (e.g. confidence intervals)
☒	☐	For null hypothesis testing, the test statistic (e.g. F, t, r) with confidence intervals, effect sizes, degrees of freedom and P value noted<br>*Give P values as exact values whenever suitable.*
☒	☐	For Bayesian analysis, information on the choice of priors and Markov chain Monte Carlo settings
☒	☐	For hierarchical and complex designs, identification of the appropriate level for tests and full reporting of outcomes
☒	☐	Estimates of effect sizes (e.g. Cohen's d, Pearson's r), indicating how they were calculated

Our web collection on statistics for biologists contains articles on many of the points above.

Software and code

Policy information about availability of computer code

Data collection

CD-HIT v4.7
SortMeRNA v2.1
USEARCH v10 (incorporates UBLAST algorithm)
MAFFT v7.0
trimAl v1.4
RaxML v8.2.9
FigTree v1.4.4
SIAS (http://imed.med.ucm.es/Tools/sias.html) version updated 10 March 2020
BWA v0.7.17 (incorporates BWA-MEM and backtrack algorithms)
PathoScope v2.0.7
SPAdes v3.5.054
BLAST+ suite v2.4
KronaTools v2.7
pHMMER online server (v2.41.1)
MFOLD online server
Circos v0.69-8
NCBI ORFfinder
SAMtools v0.1.19
BBMap v35 (incorporates BBDuk.sh)
CodonCode Aligner v9.0.1
Easyfig v2.2.564

Data analysis	R v3.6.1

For manuscripts utilizing custom algorithms or software that are central to the research but not yet described in published literature, software must be made available to editors/reviewers. We strongly encourage code deposition in a community repository (e.g. GitHub). See the Nature Research guidelines for submitting code & software for further information.

Data

Policy information about availability of data

All manuscripts must include a data availability statement. This statement should provide the following information, where applicable:

- Accession codes, unique identifiers, or web links for publicly available datasets
- A list of figures that have associated raw data
- A description of any restrictions on data availability

Viral genomes and coding sequences are available under NCBI accessions MT293410-MT293429. Raw sequencing reads are available under European Nucleotide Archive study accession PRJEB35571. Protein alignments and tree files are available from Figshare project 84065. GenBank databases are available via NCBI (https://www.ncbi.nlm.nih.gov/), and the Reference Proteome database was integrated with the pHMMER web service (https://www.ebi.ac.uk/Tools/hmmer/search/phmmer)

Field-specific reporting

Please select the one below that is the best fit for your research. If you are not sure, read the appropriate sections before making your selection.

[x] Life sciences [ ] Behavioural & social sciences [ ] Ecological, evolutionary & environmental sciences

For a reference copy of the document with all sections, see nature.com/documents/nr-reporting-summary-flat.pdf

Life sciences study design

All studies must disclose on these points even when the disclosure is negative.

Sample size	No sample size calculation was performed. We analysed all available sequencing data from individuals in two large HIV-1 cohorts (total N=374), which was considered sufficient for our study.
Data exclusions	For cohort '1984', if a sample had received more than one sequencing run - we excluded the run with the lowest yield. This was decided in advance - since usually the reason for a second run was sub-par yield in the first run.
Replication	In addition to our own cohorts, we analysed sequencing data from macaques in California and humans in South America. We were able to reproduce observed associations between viruses and parasites - although Giardia was not detected. We found the same data patterns in both our cohorts separately, despite methodological differences in library preparation.
Randomization	Allocation of samples for sequencing was random, and was carried out for an unrelated study.
Blinding	Virus and/or parasite status of samples was blinded when samples were sent for confirmatory parasitological testing in a collaborating laboratory.

Reporting for specific materials, systems and methods

We require information from authors about some types of materials, experimental systems and methods used in many studies. Here, indicate whether each material, system or method listed is relevant to your study. If you are not sure if a list item applies to your research, read the appropriate section before selecting a response.

Materials & experimental systems

n/a	Involved in the study	
x		Antibodies
x		Eukaryotic cell lines
x		Palaeontology
x		Animals and other organisms
	x	Human research participants
x		Clinical data

Methods

n/a	Involved in the study	
x		ChIP-seq
x		Flow cytometry
x		MRI-based neuroimaging

Human research participants

Policy information about studies involving human research participants

Population characteristics	The 374 subjects were from two cohorts. Cohort 1 '1994' included 194 HIV-1 infected individuals not on active antiretroviral therapy. All individuals were aged 18 or older, 12 were female, 182 were male. Cohort 2 '1984' were 180 men who have sex with men. 85 were HIV-1 positive, 95 were HIV-1 negative. Subjects were 18 or older.
Recruitment	Cohort 1 were recruited for a study into unexplained causes of diarrhoea. Subjects were recruited on visitation to an out-patient clinic at the Amsterdam Medical Center in 1994 and 1995, and were asked to bring a stool sample on their subsequent visit. Criteria were HIV-1 positivity, and aged 18 and older.<br>Cohort 2 were recruited as part of a prospective HIV-1 study among MSM, inititated in 1984.<br><br>Our cohort contained a high proportion of men, a majority of whom were HIV-1 positive MSM. MSM are known to have a higher than average infection rate with gastrointestinal parasites, including the targets of this study (Entamoeba and Giardia). This bias likely increased the number of parasite positive samples we detected, and therefore probably also the number of 'parasite-virus' positive samples. Since the number of parasite-virus samples was still low (21 of 374), this implies MSM are an ideal population in which to identify parasite associated viruses.
Ethics oversight	Medical Ethics Committee of the Amsterdam University Medical Center (MEC 07/182)

Note that full information on the approval of the study protocol must also be provided in the manuscript.
